# Variation in the LRR region of Pi54 protein alters its interaction with the AvrPi54 protein revealed by *in silico* analysis

**DOI:** 10.1371/journal.pone.0224088

**Published:** 2019-11-05

**Authors:** Chiranjib Sarkar, Banita Kumari Saklani, Pankaj Kumar Singh, Ravi Kumar Asthana, Tilak Raj Sharma

**Affiliations:** 1 ICAR-Indian Agricultural Research Institute, New Delhi, India; 2 ICAR-Indian Agricultural Statistics Research Institute, New Delhi, India; 3 ICAR-National Research Centre on Plant Biotechnology, New Delhi, India; 4 Banaras Hindu University, Varanasi, Uttar Pradesh, India; 5 National Agri-Food Biotechnology Institute, Mohali, Punjab, India; Fujian Agriculture and Forestry University, CHINA

## Abstract

Rice blast, caused by the ascomycete fungus *Magnaporthe oryzae* is a destructive disease of rice and responsible for causing extensive damage to the crop. *Pi54*, a dominant blast resistance gene cloned from rice line Tetep, imparts a broad spectrum resistance against various *M*. *oryzae* isolates. Many of its alleles have been explored from wild *Oryza* species and landraces whose sequences are available in the public domain. Its cognate effector gene *AvrPi54* has also been cloned from *M*. *oryzae*. Complying with the Flor’s gene-for-gene system, Pi54 protein interacts with AvrPi54 protein following fungal invasion leading to the resistance responses in rice cell that prevents the disease development. In the present study *Pi54* alleles from 72 rice lines were used to understand the interaction of Pi54 (R) proteins with AvrPi54 (Avr) protein. The physiochemical properties of these proteins varied due to the nucleotide level polymorphism. The *ab initio* tertiary structures of these R- and Avr- proteins were generated and subjected to the *in silico* interaction. In this interaction, the residues in the LRR region of R- proteins were shown to interact with the Avr protein. These *R* proteins were found to have variable strengths of binding due to the differential spatial arrangements of their amino acid residues. Additionally, molecular dynamic simulations were performed for the protein pairs that showed stronger interaction than Pi54^tetep^ (original Pi54 from Tetep) protein. We found these proteins were forming h-bond during simulation which indicated an effective binding. The root mean square deviation values and potential energy values were stable during simulation which validated the docking results. From the interaction studies and the molecular dynamics simulations, we concluded that the AvrPi54 protein interacts directly with the resistant Pi54 proteins through the LRR region of Pi54 proteins. Some of the Pi54 proteins from the landraces namely Casebatta, Tadukan, Varun dhan, Govind, Acharmita, HPR-2083, Budda, Jatto, MTU-4870, Dobeja-1, CN-1789, Indira sona, Kulanji pille and Motebangarkaddi cultivars show stronger binding with the AvrPi54 protein, thus these alleles can be effectively used for the rice blast resistance breeding program in future.

## Introduction

Rice is the widely consumed staple food throughout the globe. Rice is predominantly cultivated in Asia and it is the source of 23% of the calories consumed by the global human population [1]. The crop is vulnerable to a number of biotic and abiotic stresses. High losses incurred due to various diseases can threaten the global food security. Rice blast, caused by the fungus *Magnaporthe oryzae*, causes a big loss to the net production of rice [2]. Lot of research is being undertaken to explore the genomics, host-pathogen interactions, mechanism of development of disease and breeding strategies with an aim to establish effective disease management strategy. Whole genome sequences for both the organisms are available in public domain which have accelerated the efforts to identify and characterize the determinants of blast disease, both in rice and the fungus. Some determinants of the disease development are avirulence (*Avr*) genes in the pathogen which condition pathogenicity and resistance (*R*) genes in the host which condition the defense reaction in response to the pathogen invasion. Many *R* genes from rice and *Avr* genes from *M*. *oryzae* have been identified and characterized. Nearly 350 QTLs for resistance to rice blast and 101 *R* genes have been identified out of which 23 *R* genes have been molecularly characterized [3,4,5]. A total of 25 *Avr* genes of *M*. *oryzae* have been genetically mapped out of which 11 *Avr* genes have also been cloned and characterised [6]. The pathogenic races of *M*. *oryzae* which carry the dominant *Avr* gene are unable to develop disease in certain cultivars of the host species which carry the dominant cognate *R* gene. Such host plants develop defense responses following the fungal infection and restrict the disease development. These *Avr* and *R* gene follow the gene-for-gene hypothesis [7]. The hypothesis implies that the Avr proteins can be recognized directly in those cultivars of the host species which has functional protein coded by corresponding *R* genes. Such cultivars do not develop the disease. The R-Avr interaction triggers the hypersensitive cell death in the plant which kills the infected cell, thereby checking the invasion of the pathogen to non-infected cells. The interaction between the R- and Avr- proteins can even be direct through some modified proteins of the host [8, 9].

The exploitation of host plant resistance is one of the most economical and environmentally safe approaches to develop resistance against blast disease [10]. Efforts are going on for identification and the deployment of the *R* genes to develop resistant cultivars against rice blast worldwide. A dominant *R* gene, *Pi54* has been cloned from *indica* rice line ‘Tetep’ and validated to impart wide spectrum resistance in rice against diverse *M*. o*ryzae* strains [11,12,13]. It is responsible for activation of defence response genes in response to the pathogen attack [14]. It contains a zinc finger domain and an LRR domain [11,15]. The C-terminal LRR domain of the *R* genes is involved in ligand recognition and binding [16]. This interaction activates the host defense mechanism via signal cascade leading to activation of the genes involved in hypersensitive resistance response [3]. LRR region exhibits more variations than other regions of the gene [17]. Orthologues of *Pi54* named as *Pi54of* and *Pi54rh* that confer high degree of resistance to *M*. *oryzae* were cloned from wild species of rice [18,19]. The *in silico* protein modelling and molecular docking between Pi54 protein and candidate effector proteins from *M*. *oryzae* have been used as a strategy to find the probable avirulent *AvrPi54* gene by analyzing the interaction at the LRR domain of the Pi54 protein. *AvrPi54* has then been cloned and characterised [20]. The molecular docking was also used to display interaction of *Pi54of* protein with *Avr-Pi54* through STI1 and RhoGEF domains which are components of the rice defensome complex [19].

The Pi54 and AvrPi54 interactions are essential to understand the mechanism of blast disease development. There are many allelic variants of the Pi54 resistance gene explored from various landraces and are reported to have unique polymorphic patterns at nucleotide level [16]. The sequence polymorphism in these alleles can result in structural variation at the protein level which can alter the interacting surface and impact the interaction potential with AvrPi54 protein. Our present study has analyzed the impact of nucleotide level polymorphism among Pi54 alleles and on their properties, structure and interaction with AvrPi54 protein. We have determined structures of 72 allelic Pi54 proteins and studied their interaction with the AvrPi54 protein by molecular docking using *in silico* tools, aimed to find more suitable alleles which can be used in breeding programs to develop blast resistance in rice. The online available bioinformatics tools for protein modelling and interaction have been used in this study [21]. Thus it saves the time and cost of the experimental structure determinations by X-Ray Crystallography and nuclear magnetic resonance. The most efficient docking algorithms often produce models with atomic-level accuracy. Molecular docking is widely used in drug discovery as it is one of the most reliable methods for the prediction of the interaction between two molecules [22]. It allows the assessment of the key residues located at the active site of the target molecule that participates in interaction with the ligand. The approach has been extended to the R-Avr interaction analysis. [19,20,23]. The alleles whose protein products show more interaction with the AvrPi54 protein can further be examined experimentally and deployed in the development of more resistant cultivars.

## Materials and methods

### Sequence retrieval

The nucleotide sequence of the *AvrPi54* gene with the accession number HF545677 was retrieved from European Nucleotide Archive (ENA) of European Molecular Biology Laboratory (EMBL) Nucleotide Sequence Database. The nucleotide sequence of the blast resistance gene *Pi54* (*Pi54*^*tetep*^), (Accession no. AY914077) was retrieved from NCBI gene database (www.ncbi.nlm.nih.gov/). The nucleotide sequence of 72 alleles of *Pi54* from different land races of rice has been retrieved from the EMBL database which is listed in S1 Table.

### Domain identification in Pi54 proteins

The amino acid sequence of all the *Pi54* alleles were deduced from the nucleotide sequences using the web based tool FGENESH (http://linux1.softberry.com/berry). The monocot plants (Corn, Rice wheat, Barley) were selected under organism specific gene-finding parameters. The LRR domain was identified in these allelic proteins by aligning with the already predicted LRR domain of the Pi54 protein originally cloned from rice variety Tetep as described earlier [11].

### Multiple sequence alignment of the LRR region

The nucleotide sequences of the LRR region of *Pi54* alleles were aligned with the nucleotide sequence of *Pi54* gene from Tetep one by one using the online web-based tool Clustal omega (http://www.ebi.ac.uk/Tools/msa/clustalo/). The output file of the Clustal omega was saved in FASTA format and analysed using DNASP (DNA sequence polymorphism) standalone software [24] to identify SNPs and InDels. Amino acid substitutions and frame shifts in these Pi54 proteins were determined by the Clustal omega software (http://www.ebi.ac.uk/Tools/msa/clustalo/). The output of the Clustal omega tool was viewed in JalView [25] to identify amino acid substitution and conserved regions in the alignment.

### Determination of physiochemical properties of various Pi54 proteins

The primary structure of the Pi54 proteins was studied using ProtParam tool (http://web.expasy.org/protparam/) of Expasy Server and molecular weight, isoelectric point, instability index, aliphatic index, and grand average hydropathy (GRAVY) were computed.

### Tertiary structure (3D) prediction and modelling of Pi54 proteins

All the protein sequences of the Pi54 alleles used in this study showed less than 10% identity in similarity search against Protein Data Bank [26] (http://www.rcsb.org/pdb/home/home.do) using the BLAST tool [27]. Therefore, their tertiary structure (3D) prediction was done with *ab initio* based protein modelling procedure using the web-based server I-TASSER (Iterative Threading Assembly Refinement) (http://zhanglab.ccmb.med.umich.edu/I-TASSER/). Each predicted protein structure was visualized in RasMol visualization tool [28] and the numbers of different secondary structures like alpha-helix, beta-sheet, turns, coil and total numbers of hydrogen bonds etc. were calculated. The 3D structures of Pi54 proteins were assessed by Ramachandran plot [29] in Discovery Studio 2.0 (Accelrys Life Science Tool). Refinement of structure was done for those models that showed residues below the expected value (~98%) in favourable region in Ramachandran plot using the ModRefiner server (http://zhanglab.ccmb.med.umich.edu/ModRefiner/) and the residues in favourable region were increased. The energy minimization of the refined models was performed by CHARMM 19 (Chemistry at HARvard Macromolecular Mechanics) Force Field [30]. These procedures of structure refinement and energy minimization were iterated till the optimized structures were obtained. Similar strategy was employed to get the tertiary structure of mature AvrPi54 protein after cleaving the signal peptide region of 19 amino acids. The signal peptide sequence was predicted with TargetP tool (http://www.cbs.dtu.dk/services/TargetP/).

### Quantitative similarity assessment of protein structures

The similarity of the various Pi54 protein structures were compared with the Pi54^tetep^ protein using the TM-align tool (http://zhanglab.ccmb.med.umich.edu/TM-align/). This tool performs the structural comparison and residue-to-residue alignment. The optimal superposition of two protein structure gives the TM-score value and RMSD value. The TM-score was calculated for the residue pairs of the two proteins within 0.5 Å atomic distances.

### Molecular docking of Pi54 and AvrPi54 proteins

Each Pi54 protein and AvrPi54 protein pair was subjected to molecular docking by the standalone Z-Dock software available in the Discovery Studio 2.0 (Accelrys Life Science Tool). This method performs calculation of docked protein poses, filtering of docked protein poses, re-rank docked protein poses and cluster docked protein poses and calculation of cluster density. The docking was first performed between the Pi54^tetep^ protein and the AvrPi54 protein. The result of the interaction analysis of the docking of Pi54^tetep^ protein and the AvrPi54 protein was used as a control and same parameters were used for other docking analysis (Table 1). The best docked pose for each pair was thus determined and used for further analysis.

**Table 1:** The parameters used for performing docking in Z-Dock software.

Parameters	Value
Angular step size	6
Distance cut-off	10.0
ZRank	True
Zrank Top poses	10
Clustering Top poses	10
Clustering RMSD cut-off	10.0
Clustering Interface cut-off	10.0
Maximum number of clusters	2
Parallel processing	False
Parallel processing server order	True
Use electrostatic and desolvation energy	True

### Calculation of binding energy

Depending on the docking results of Pi54 and AvrPi54 proteins the binding energy of the interaction were calculated for the interacted pairs. The binding energy depends on the interacting residues of the two proteins and the atoms participating in the interaction.

The energy difference was calculated using the equation:

QE=Ecomplex-Eligand-Eprotein(QEistheligandbindingenergy)

### Molecular dynamic simulation

In order to assess the reliability of the docking results and to understand their stability, molecular dynamics simulations was performed for the chosen poses of a few pairs of proteins under our study from the docking results by using GROMACS version 2018.1 [31] with the force field as GROMOS96 54a7 force field [32]. The docked structures with minimum binding energy and maximum interaction values were chosen and executed for 100 ps long MD simulations, and conformations were saved at 0.001 ps intervals. The proteins structures of each pair were solvated, minimized and equilibrated. The solvation was done with spc216 water model in a cubic box (10.4 × 10.4 × 10.4 nm^3^) and the Counter-ion (Na^+^) was included to counterbalance the solvated system. To minimise the steric hindrances in the solvated system of protein–ligand complex, energy minimization was done using the steepest algorithm up to a maximum 50,000 steps or until the maximum force (Fmax) did not exceed the default threshold of 1000 kJ mol^-1^ nm^-1^. The system was first equilibrated using NVT ensemble followed by NPT ensemble for 50,000 steps (100 ps) at 300 K temperature and 1 atm pressure. The molecular dynamic simulations were carried out for 2 ns long and the Root Mean Square Deviation (RMSD), Root Mean Square Fluctuation (RMSF), hydrogen bonds and energy plots were generated.

## Results

### Physio-chemical properties of Pi54 proteins

The amino acid sequences for all the alleles were analyzed and compared to the *Pi54*^*tetep*^. The size of the Pi54 proteins under study varied from 173 amino acids to 486 amino acids while the Pi54^tetep^ protein is 330 amino acids long. Their molecular weights were quite variable due to the change in number of amino acid residues in the proteins. These proteins were found to have high content of some amino acids such as leucine, glutamic acid and cysteine. Maximum percentage of Leucine in these proteins was nearly 17% (S1 Fig). Their physio-chemical properties like theoretical pI, GRAVY, aliphatic index and molecular weight (Table 2) showed variation from the Pi54^tetep^ protein. All the proteins except Dobeja-1, ND 118 and Samleshwari were acidic as they had pI values in the acidic range. The aliphatic index of a protein is an indicator of the relative volume occupied by aliphatic side chains of amino acids such as alanine, valine, leucine, and isoleucine. It measures thermal stability of the proteins [33]. Aliphatic index was high for most of the proteins. The GRAVY value of the Pi54^tetep^ protein was negative, *i*.*e*. -0.054 which indicates its good affinity for water. The GRAVY value of proteins in this study had both negative and positive values. GRAVY values for all the *Pi54* proteins were observed to be greater than Pi54^tetep^ protein except the alleles derived from Bidarlocal-2, Casbatta, Indrayani, IR-64, PR-118, Tadukan, Varun dhan and HR-12. The GRAVY values showed very large variation in case of the proteins like Basmati-386, HPR-2083, HPR-2178, IRBB-55, IRBB-4, Jatto, Kulanji Pille, LD-43 and Tiyun.

**Table 2:** The physio-chemical properties of the Pi54 proteins.

Rice lines	Theoretical pI	Molecular weight (kDa)	Leucine %	GRAVY Index	Aliphatic index
Tetep	5.00	37.30	16.70	-0.05	104.00
Acharmita	6.31	27.38	17.50	0.14	105.62
Basmati 386	5.12	51.92	17.40	0.01	105.57
Belgaum basmati	5.12	43.09	17.30	0.08	108.24
Bidarlocal-2	4.91	21.54	16.60	-0.09	102.59
Budda	6.31	31.84	16.20	0.14	101.69
Casbatta	5.12	19.39	16.80	-0.20	98.67
Chiti zhini	5.39	45.38	17.50	0.06	107.21
CN-1789	5.18	30.63	17.10	0.06	106.95
CSR 10	5.34	45.26	17.20	0.04	104.31
CSR-60	5.12	43.09	17.30	0.08	108.24
Dobeja-1	8.35	32.25	17.00	0.25	105.41
Gonrra bhog	5.18	43.45	16.30	0.13	107.19
Govind	5.59	41.96	15.50	0.14	101.17
Gowrisanna	5.06	39.22	14.50	0.07	102.56
Himalya 799	5.55	50.02	17.20	0.06	105.87
HLR-108	5.12	43.10	17.30	0.08	108.24
HLR-142	5.12	43.10	17.30	0.08	108.24
HPR 2083	5.07	40.20	16.90	0.02	105.46
HPR-2178	5.25	28.12	17.30	-0.02	107.34
HR-12	4.82	23.31	16.30	-0.11	103.59
IC356437	5.12	43.10	17.30	0.08	108.24
Indira sona	5.51	50.13	17.20	0.05	104.33
Indrayani	5.93	47.36	16.70	-0.07	102.60
INRC 779	5.18	30.63	17.10	0.06	106.95
IR 64	4.84	21.63	16.70	-0.08	102.59
IRAT-144	5.07	40.37	17.50	0.05	108.20
IRBB 55	5.45	54.30	17.70	-0.02	104.81
IRBB-13	5.12	43.09	17.30	0.08	108.24
IRBB-4	5.16	54.85	16.90	-0.01	103.13
Jatto	5.64	49.98	17.10	0.01	103.05
Kari kantiga	5.12	43.10	17.30	0.08	108.24
Kariya	5.12	43.10	17.3	0.08	108.24
Kasturi	5.33	47.78	17.10	0.06	106.06
Kavali kannu	6.31	27.39	17.50	0.14	105.62
Kulanji pille	5.44	43.73	15.00	-0.01	98.77
LD-43 (HLR-144)	5.24	42.45	17.60	0.01	107.07
Lalnakanda	5.12	43.10	17.30	0.08	108.24
Mahamaya	5.34	45.26	17.20	0.04	104.31
Malviya dhan	5.38	39.15	17.40	0.04	104.30
Mesebatta	5.18	30.63	17.10	0.06	106.95
Mote bangarkaddi	5.18	30.63	17.10	0.06	106.95
MTU 4870	5.93	48.82	14.60	0.06	100.00
MTU-1061	5.09	43.11	16.50	0.04	107.98
ND 118	8.33	35.21	17.40	0.10	101.58
Orugallu	5.13	40.26	16.90	0.02	105.46
Pant sankar dhan 1	5.12	43.10	17.30	0.08	108.24
Pant sankar dhan 17	5.67	40.32	18.00	-0.03	104.63
Parijat	5.32	45.36	17.70	0.07	108.18
Parimala kalvi	5.39	45.38	17.50	0.06	107.21
PR 118	4.98	21.58	16.60	-0.10	102.07
Pusa 33	5.48	41.56	17.40	0.04	106.78
Pusa basmati 1	5.27	45.32	17.20	0.03	104.06
Pusa Sugandh 3	5.12	43.10	17.30	0.08	108.24
Pusa Sugandh 4	5.12	43.10	17.30	0.08	108.24
Ram Jawain 100	5.14	40.32	16.90	0.04	106.85
Ranbir basmati	5.12	43.10	17.30	0.08	108.24
Sadabahar	5.32	45.36	17.70	0.07	108.18
Samleshwari	8.64	27.12	17.50	0.12	105.62
Sanna mullare	5.18	30.63	17.10	0.06	106.95
Sathia -2	5.24	43.22	17.40	0.03	106.61
Satti	5.34	45.26	17.20	0.04	104.31
Shiva	5.63	43.06	15.10	0.09	94.63
Superbasmati	5.12	43.10	17.30	0.08	108.24
Suphala	5.32	45.36	17.70	0.07	108.18
T23	5.33	47.66	17.10	0.08	106.31
Tadukan	4.78	21.54	16.60	-0.09	102.59
Taipei-309	5.18	30.63	17.10	0.06	106.95
Thule ate	5.63	43.06	15.10	0.09	94.63
Tilak chandan	5.12	43.10	17.30	0.08	108.24
Tiyun	5.69	36.08	16.90	-0.01	104.20
V L Dhan	6.31	27.38	17.50	0.14	105.62
Vanasurya	5.18	30.63	17.10	0.06	106.95
Varalu	5.32	45.36	17.70	0.07	108.18
Varun dhan	5.19	35.51	16.00	-0.07	101.57

### Analysis of LRR region in Pi54 proteins

**Fig 1:**
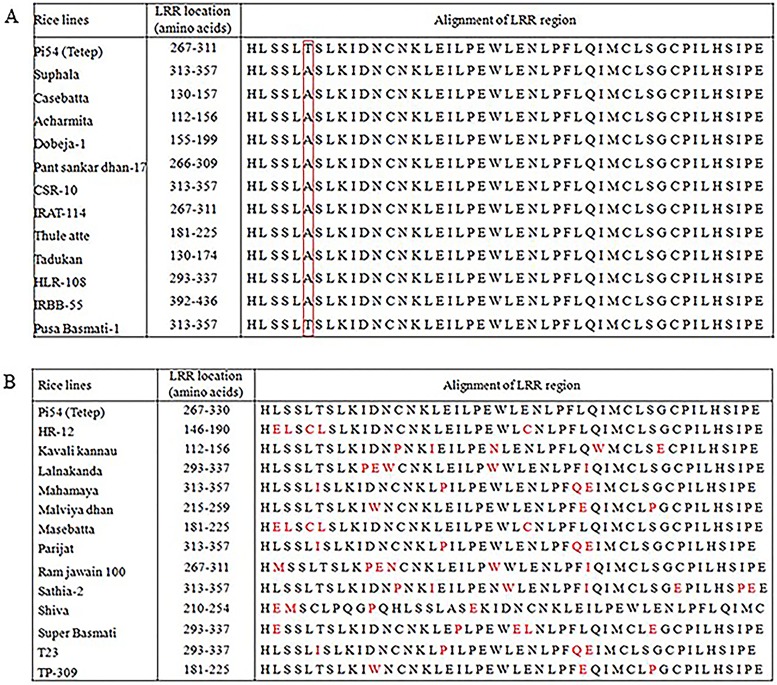
Alignment of LRR region of the Pi54 proteins. **(A)** shows conservation and **(B)** shows variation in LRR region using few representative proteins. Pi54^tetep^ protein used as control.

**Fig 2:**
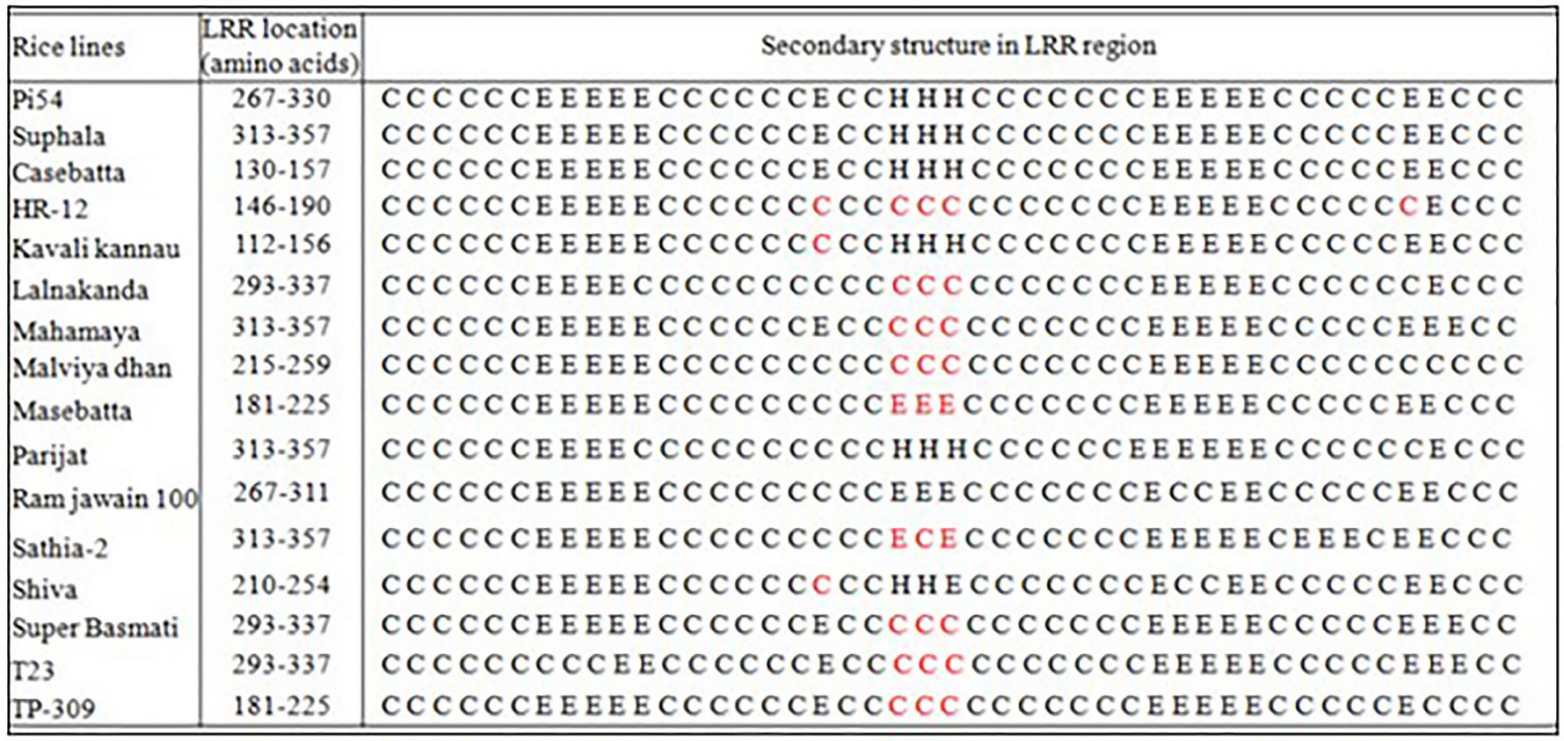
Comparison of secondary structural elements in the LRR region of the Pi54 proteins to the Pi54^tetep^ protein. Secondary structural elements in some of the Pi54 proteins were conserved whereas but varied in few other proteins having diverse LRR region when compared to Pi54^tetep^ protein. C- coils, E- β-sheet, H- helices.

A stretch of 45 amino acids from 267 to 311 amino acids has been predicted as the LRR region in Pi54^tetep^ protein [11]. The LRR region in all the Pi54 proteins was predicted by their sequence alignment to the Pi54^tetep^ protein. The amino acid sequences of the Pi54 proteins from some cultivars showed high conservation in LRR region (Fig 1A), while in some others, LRR region contained more number of substitutions (Fig 1B). The average leucine percentage and the number and arrangements of alpha-helix, beta-sheets and turns in some of the Pi54 proteins having conserved LRR region were similar to that of Pi54^tetep^ protein, whereas these protein features varied for the other proteins having diverse LRR region (Fig 2).

### Tertiary structure prediction and modelling of the Pi54 proteins

**Fig 3:**
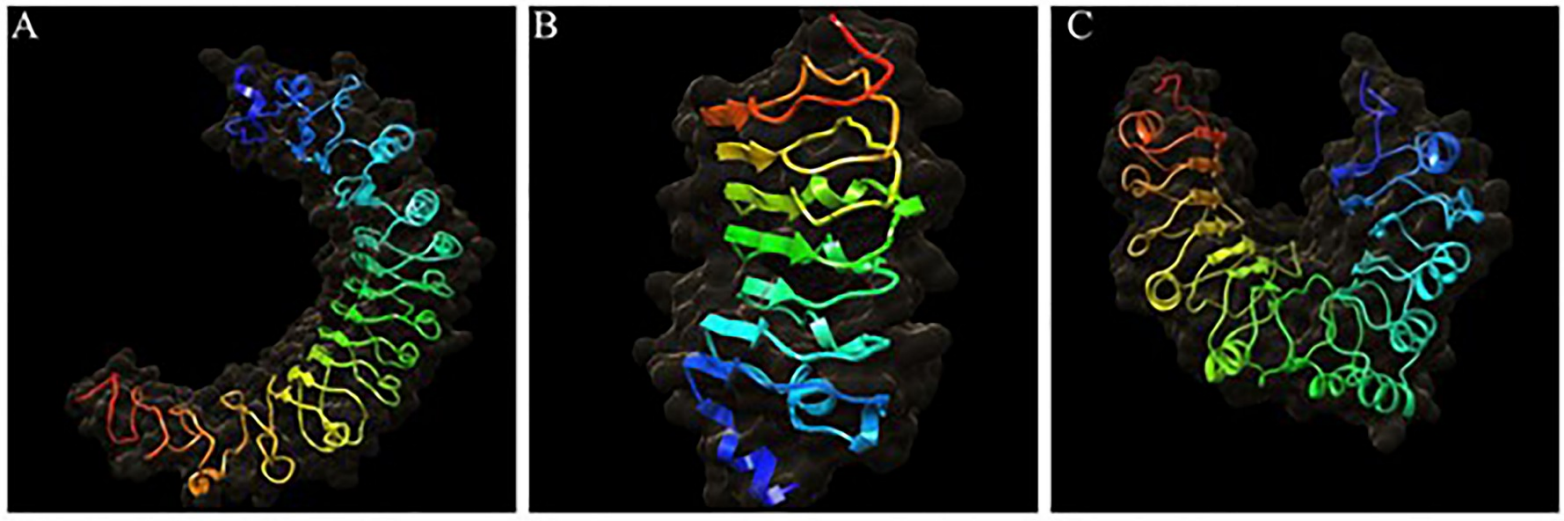
Three dimensional structures of Pi54 proteins. Some of R proteins showed a typical horseshoes shaped structure but such geometry was not seen in all the Pi54 proteins. 3D structures from rice lines, A-Tetep, B- Casbatta, C- HPR– 2083.

The three-dimensional (3D) structures of all the Pi54 proteins are given in Fig 3 and S2 Fig. The Pi54^tetep^ protein showed a typical horseshoes shaped structure but such geometry was not seen in all the Pi54 proteins. The horseshoe shaped structures were obtained for 36 proteins. Each protein molecule showed a motif having a curved region lined with the parallel beta strands on inner side and all the alpha helices on the one side of the beta sheet. The α-helices and β-sheets folded into tertiary structure and they were stabilized by hydrogen bonds. In case of the Pi54^tetep^ protein the number of helices, strands and turns were predicted as 9, 17 and 53, respectively, and total 244 H-bonds were also identified in this protein. The numbers of helices, strands and turns varied in different Pi54 proteins (Table 3). The potential energy, vander waals energy, electrostatic energy and sum of all the total energy, *i*.*e*. global free minimum energy were calculated for all Pi54 proteins (Table 4). The total energy of Pi54^tetep^ protein is -37033.6752kcal/mol. The global free minimum energy of the Pi54 proteins showed that some of these proteins from rice lines like Kasturi, Kulanji pille, HLR-144, Mahamaya, ND 118 etc. had lesser energy than the Pi54^tetep^.

**Table 3:** Secondary structures of the Pi54 proteins.

Rice lines	Helices	Strands	Turns	Rice lines	Helices	Strands	Turns
Tetep	9	17	53	Lalnakanda	14	18	66
Acharmita	3	17	29	Mahamaya	11	26	65
Basmati 386	14	30	85	Malviya dhan	3	21	61
Belgaum basmati	14	18	66	Mesebatta	6	15	39
Bidarlocal-2	4	15	46	Mote bangarkaddi	5	24	41
Budda	11	23	40	MTU 4870	10	25	45
Casbatta	8	13	25	MTU-1061	12	19	58
Chiti zhini	14	22	66	ND 118	13	18	42
CN-1789	8	19	42	Orugallu	9	26	61
CSR 10	13	34	58	Pant sankar dhan 1	13	34	58
CSR-60	14	18	66	Pant sankar dhan 17	11	31	49
Dobeja-1	8	14	47	Parimala kalvi	14	22	66
Gonrra bhog	14	19	57	Parijat	13	25	58
Govind	12	20	56	PR 118	8	18	43
Gowrisanna	10	28	56	Pusa basmati 1	13	21	67
Himalya 799	12	31	61	Pusa Sugandh 3	13	34	58
HLR-108	13	34	58	Pusa Sugandh 4	14	18	66
HLR-142	13	34	58	Ram Jawain 100	6	28	55
HPR 2083	12	20	56	Ranbir basmati	14	18	66
HPR-2178	11	17	32	Sadabahar	10	27	66
HR-12	5	19	28	Samleshwari	4	17	39
IC356437	14	18	66	Sanna mullare	6	15	39
Indira sona	14	27	68	Sathia -2	10	19	60
Indrayani	8	19	76	Satti	13	34	58
INRC 779	6	15	39	Shiva	8	19	35
IR 64	9	10	53	Superbasmati	14	18	66
IRAT-144	14	24	55	Suphala	13	33	56
IRBB 55	17	30	76	T23	14	18	66
IRBB-13	13	34	58	Tadukan	3	13	29
IRBB-4	13	42	89	Taipei-309	6	15	39
Jatto	15	22	66	Thule ate	17	21	57
Kari kantiga	14	18	66	Tilak chandan	14	18	66
Kariya	14	18	66	Tiyun	10	21	50
Kasturi	14	18	66	V L Dhan	6	6	34
Kavali kannu	2	15	38	Vanasurya	6	15	39
Kulanji pille	10	23	45	Varalu	10	27	66
LD-43 (HLR-144)	15	15	55	Varun dhan	7	13	47

**Table 4:** Energy parameters (kcal/mol) of the Pi54 proteins calculated by the CHARMm force field.

Rice lines	Potential Energy	Van der waals Energy	Electrostatic Energy	Total Energy
Tetep	-17820.86	-2601.34	-16611.47	-37033.67
Acharmita	-19734.10	-2938.24	-18377.58	-41049.93
Basmati 386	-19706.05	-2909.36	-18377.58	-40993.01
Belgaum basmati	-19639.22	-2909.36	-18377.58	-40926.17
Bidarlocal-2	-19693.89	-2909.36	-18336.75	-40940.01
Budda	-19520.04	-2909.36	-18236.06	-40665.47
Casbatta	-19520.04	-2887.16	-18236.06	-40643.27
Chiti zhini	-19514.89	-2887.16	-18236.06	-40638.12
CN-1789	-19514.89	-2887.16	-18236.06	-40638.12
CSR 10	-19514.89	-2887.16	-18155.40	-40557.45
CSR-60	-19514.89	-2885.53	-18155.40	-40555.82
Dobeja-1	-19392.08	-2864.14	-18089.12	-40345.36
Gonrra bhog	-18685.14	-2864.14	-17509.05	-39058.35
Govind	-18685.14	-2864.14	-17309.29	-38858.58
Gowrisanna	-18685.14	-2864.14	-17309.29	-38858.58
Himalya 799	-18685.14	-2864.14	-17309.29	-38858.58
HLR-108	-18685.14	-2864.14	-17309.29	-38858.58
HLR-142	-18685.14	-2864.14	-17309.29	-38858.58
HPR 2083	-18685.14	-2864.14	-17309.29	-38858.58
HPR-2178	-18685.14	-2864.14	-17309.29	-38858.58
HR-12	-9608.120	-1391.91	-9103.56	-20103.61
IC356437	-18685.14	-2864.14	-17309.29	-38858.58
Indira sona	-18685.14	-2864.14	-17309.29	-38858.58
Indrayani	-18685.14	-2864.14	-17309.29	-38858.58
INRC 779	-18685.14	-2864.14	-17309.29	-38858.58
IR 64	-18685.14	-2864.14	-17309.29	-38858.58
IRAT-144	-18685.14	-2864.14	-17309.29	-38858.58
IRBB 55	-18685.14	-2839.64	-17309.29	-38834.08
IRBB-13	-18651.39	-2809.70	-17309.29	-38770.38
IRBB-4	-18425.21	-2747.20	-17027.72	-38200.14
Jatto	-18261.65	-2699.01	-17022.62	-37983.29
Kari kantiga	-17820.86	-2699.01	-16611.47	-37131.34
Kariya	-17792.63	-2699.01	-16597.36	-37089.02
Kasturi	-17792.63	-2648.10	-16511.46	-36952.19
Kavali kannu	-9639.09	-1391.10	-9034.47	-20065.48
Kulanji pille	-17792.63	-2641.98	-16511.46	-36946.07
LD-43 (HLR-144)	-17723.68	-2641.98	-16511.46	-36877.12
Lalnakanda	-9627.86	-1391.91	-9034.48	-20054.26
Mahamaya	-17657.51	-2641.98	-16458.20	-36757.69
Malviya dhan	-9627.86	-1375.08	-9034.47	-20037.43
Mesebatta	-9627.86	-1375.08	-9034.47	-20037.43
Mote bangarkaddi	-17657.51	-2641.98	-16448.72	-36748.21
MTU 4870	-17657.51	-2608.89	-16448.72	-36715.12
MTU-1061	-17657.51	-2601.34	-16448.72	-36707.57
ND 118	-17646.81	-2589.72	-16448.72	-36685.25
Orugallu	-17155.67	-2556.39	-15947.23	-35659.29
Pant sankar dhan 1	-16146.68	-2425.20	-15026.83	-33598.73
Pant sugandh dhan17	-16096.46	-2421.34	-14874.21	-33392.02
Parijat	-9627.86	-1375.08	-9021.24	-20024.19
Parimala kalvi	-15682.81	-2392.41	-14519.86	-32595.08
PR 118	-13556.46	-2012.44	-12629.43	-28198.34
Pusa basmati 1	-13408.05	-1990.56	-12439.45	-27838.06
Pusa Sugandh 3	-13408.05	-1990.56	-12439.45	-27838.06
Pusa Sugandh 4	-13135.24	-1927.95	-12299.06	27362.26
Ram Jawain 100	-9595.74	-1375.08	-9021.24	-19992.06
Ranbir basmati	-8884.29	-1310.59	-9021.24	-19216.13
Sadabahar	-13135.24	-1927.95	-12299.06	-27362.26
Samleshwari	-13135.24	-1927.95	-12299.06	-27362.26
Sanna mullare	-13135.24	-1927.95	-12299.06	-27362.26
Sathia -2	-7249.56	-1212.63	-8334.84	-16797.02
Satti	-6196.61	-914.87	-5776.11	-12887.58
Shiva	-6196.61	-914.87	-5776.11	-12887.58
Superbasmati	-6191.19	-890.55	-5762.24	-12843.98
Suphala	-13135.24	-1927.95	-12299.06	-27362.26
T23	-6191.19	-854.72	-5762.24	-12808.16
Tadukan	-13135.24	-1927.95	-12299.06	-27362.26
Taipei-309	-6172.576	-854.72	-5744.16	-12771.46
Thule ate	-13135.24	-1927.95	-12299.06	-27362.26
Tilak chandan	-13135.24	-1927.95	-12299.06	-27362.26
Tiyun	-13135.24	-1927.95	-12299.06	-27362.26
V L Dhan	-12548.91	-1745.78	-11742.05	-26036.73
Vanasurya	-12128.76	-1717.66	-11500.36	-25346.78
Varalu	-11999.71	-1684.68	-11297.70	-24982.09
Varun dhan	-9639.092	-1607.28	-9470.56	-20716.93

The quantitative assessment of similarity of 3D structures was done by determining TM-score and RMSD values for each pair of Pi54 protein and the Pi54^tetep^ protein. The data is given in Table 5. The superposition of the two proteins was generated by the residue-to-residue alignments. The TM-align tool gives scores between 0 and 1, the TM-score < 0.2 indicated no similarity between two structures and a TM-score > 0.5 means the structures share the same fold. The Pi54 protein from the cultivar Orugallu had TM-score 0.6158 hence shared same fold as that of the Pi54^tetep^ protein and also had highest number (183) of identical residues. Proteins from Acharmita, V L Dhan and Shiva showed no similarity to the Pi54^tetep^ protein as their TM-scores were < 0.2. The proteins of HPR-2083 and IRAT-144 contained 124 and 113 identical residues, respectively. The Pi54 protein showed RMSD value between 3 and 4.

**Table 5:** The TM-score and RMSD values of alignment of Pi54 proteins to the Pi54^tetep^ protein.

Rice lines	TM-score (<5Å)	Identical residues (<5 Å)	RMSD (Å)	Rice lines	TM-score (<5Å)	Identical residues (<5 Å)	RMSD (Å)
Acharmita	0.19	26	3.44	Mahamaya	0.33	56	3.37
Basmati 386	0.24	44	3.25	Malviya dhan	0.31	50	3.63
Belgaum basmati	0.21	41	3.13	Mesebatta	0.28	47	3.63
Bidarlocal-2	0.22	38	3.36	Mote bangarkaddi	0.32	47	3.46
Budda	0.23	31	3.34	MTU 4870	0.34	61	3.65
Casbatta	0.20	40	3.27	MTU-1061	0.33	51	3.4
Chiti zhini	0.30	46	3.58	ND 118	0.30	62	3.34
CN-1789	0.31	56	3.68	Orugallu	0.61	183	3.32
CSR 10	0.34	64	3.57	Pant sankar dhan 1	0.28	47	3.63
CSR-60	0.21	41	3.13	Pant sugandh dhan 17	0.34	67	3.59
Dobeja-1	0.28	41	3.43	Parijat	0.341	66	3.58
Gonrra bhog	0.31	50	3.73	Parimala kalvi	0.30	46	3.58
Govind	0.36	62	3.63	PR 118	0.21	36	3.54
Gowrisanna	0.33	51	3.81	Pusa 33	0.26	41	3.39
Himalya 799	0.27	42	3.59	Pusa basmati 1	0.23	36	3.44
HLR-108	0.34	64	3.57	Pusa Sugandh 3	0.28	47	3.63
HLR-142	0.34	64	3.57	Pusa Sugandh 4	0.20	41	3.13
HPR 2083	0.47	124	3.3	Ram Jawain 100	0.44	104	3.27
HPR-2178	0.27	51	3.55	Ranbir basmati	0.21	41	3.13
HR-12	0.21	40	3.27	Sadabahar	0.34	66	3.58
IC356437	0.21	41	3.13	Samleshwari	0.22	41	3.34
Indira sona	0.31	48	3.54	Sanna mullare	0.28	47	3.63
Indrayani	0.37	69	3.55	Sathia -2	0.35	78	3.55
INRC 779	0.28	47	3.63	Satti	0.34	64	3.57
IR 64	0.34	68	3.34	Shiva	0.20	29	3.34
IRAT-144	0.46	113	3.33	Superbasmati	0.21	41	3.13
IRBB 55	0.25	38	3.32	Suphala	0.34	66	3.58
IRBB-13	0.28	47	3.63	T23	0.20	41	3.13
IRBB-4	0.24	36	3.55	Tadukan	0.21	35	3.39
Jatto	0.27	47	3.63	Taipei-309	0.28	47	3.63
Kari kantiga	0.21	41	3.13	Thule ate	0.31	51	3.61
Kariya	0.21	41	3.13	Tilak chandan	0.21	41	3.13
Kasturi	0.21	41	3.13	Tiyun	0.34	58	3.61
Kavali kannu	0.20	37	3.13	V L Dhan	0.17	26	3.1
Kulanji pille	0.37	58	3.63	Vanasurya	0.28	47	3.63
LD-43 (HLR-144)	0.26	38	3.59	Varalu	0.34	66	3.58
Lalnakanda	0.21	41	3.13	Varun dhan	0.30	53	3.5

### Interaction of Pi54 and Avr-Pi54 proteins

**Fig 4:**
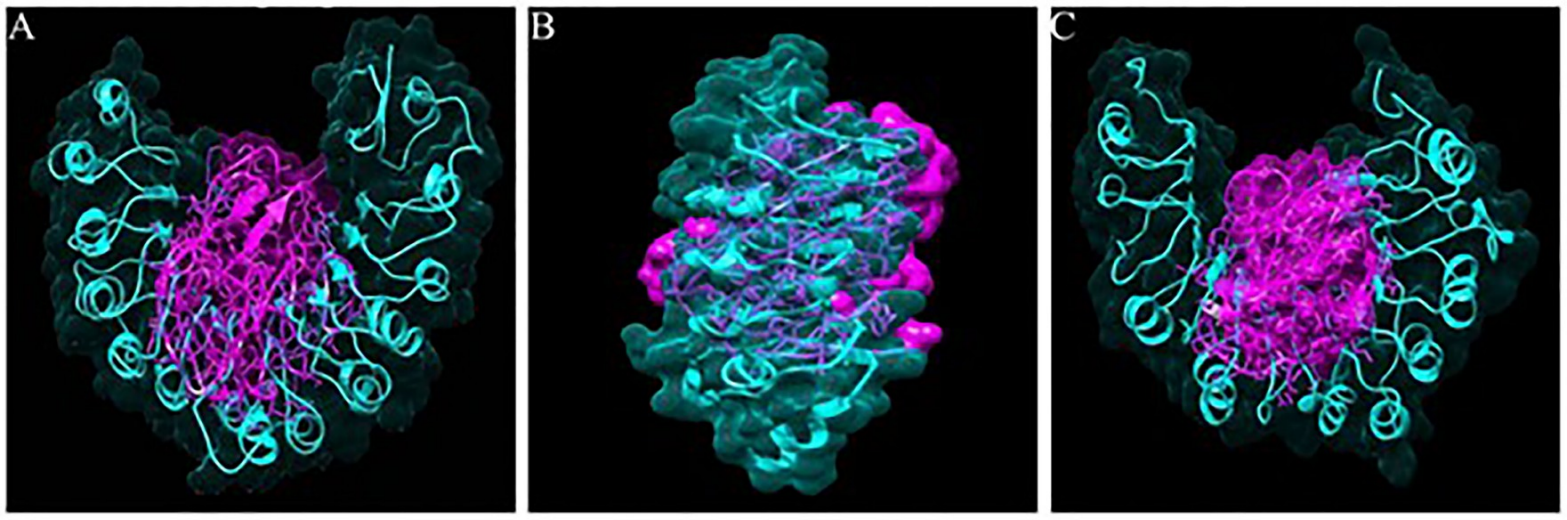
Docking images showing interaction of Avr-Pi54 and Pi54 proteins. Interaction with AvrPi54 with R proteins from rice lines, A-Tetep, B- Casbatta, C- HPR -2083.

Top ten poses of the interactions were obtained by using Discovery studio 2.0. The best pose of interaction was selected depending on the Z-dock score. Total 59 proteins showed significant interaction with Avr-Pi54 proteins. The interaction images generated are given in Fig 4 and S3 Fig. There were 13 proteins which belonged to rice lines HR12, Mesebetta, Shiva, Mahamaya, Parijat, Malviya Dhan, Ram Jawain 100, Lalankanda, Ranbir basmati, Sathia-2, Satti, Superbasmati and TP-309 did not interact with Avr-Pi54. For the interaction of Pi54 and Avr-Pi54 proteins, the H bond length less than 4Å was considered significant. In the cases of the proteins that did not show significant interaction, there were no H-bonds found within 4Å range and large separation was observed between these proteins pairs. The intermolecular interactions of the Pi54 proteins and the Avr-Pi54 protein showed that residues of the LRR domain of the Pi54 proteins participated in the interaction (Table 6). The intermolecular H-bonds were considered for the interaction analysis. More number of H-bonds was formed by the proteins of Casbatta, Gowrisanna and HLR-142 rice lines than the Pi54^tetep^ during the interaction.

**Table 6:** Intermolecular interaction of the Pi54 proteins with Avr-Pi54 protein.

Rice lines	LRR region (bp)	Interacting residues from LRR region	H-bond length (Å)	Interacting atoms	Total numbers of interaction
Tetep	267–311	LEU297-THR32	1.00	HN-O	19
Acharmita	112–156	THR133 -ALA129	1.20	HN-O	15
Basmati 386	372–416	THR389—THR112	1.13	HN-O	11
Belgaum basmati	293–337	GLY312—ILE7	1.95	HN-O	17
Bidarlocal-2	112–156	SER150—GLU31	1.07	HN-O	19
Budda	150–194	ASN175—GLY56	1.09	HN-O	12
Casbatta	130–157	GLU146—ILE10	1.44	HN-O	23
Chiti zhini	313–357	CYS345 –LEU11	1.45	HN-O	19
CN-1789	200–244	ALA219—ILE19	1.33	HN-O	15
CSR 10	313–357	LYS325—ALA34	1.36	HN-O	13
CSR-60	293–337	THR310 –ILE10	1.38	HN-O	14
Dobeja-1	155–199	ALA171—ILE7	1.88	HN-O	10
Gonrra bhog	293–337	LEU312 –SER9	1.38	HN-O	11
Govind	240–236	SER230 –TYR124	1.10	HN-O	18
Gowrisanna	192–235	ILE229—MET1	1.49	HN-O	21
Himalya 799	313–357	SER336—ALA32	2.36	HN-O	13
HLR-108	293–337	TYR318—ALA14	1.34	HN-O	14
HLR-142	293–337	THR295– GLU127	1.13	HN-O	21
HPR 2083	267–311	LYS285—ALA34	1.14	HN-O	12
HPR-2178	258–304	CYS266 –THR13	1.46	HN-O	10
IC356437	293–337	ARG296—THR37	1.48	HN-O	14
Indira sona	313–357	THR329 –SER9	1.35	HN-O	16
Indrayani	313–357	ILE345 –ILE8	1.66	HN-O	12
INRC 779	181–225	SER224—ARG33	2.03	HN-O	17
IR 64	130–174	ASN149—GLY56	1.90	HN-O	13
IRAT-144	267–311	GLU279 –GLN2	1.90	HN-O	15
IRBB 55	392–436	ALA401—ILE8	1.90	HN-O	18
IRBB-13	293–337	ILE318 –ILE7	1.64	HN-O	14
IRBB-4	398–442	MET425—VAL59	2.05	HN-O	17
Jatto	157–201	LYS199—PRO60	1.94	HN-O	12
Kari kantiga	293–337	LYS319—LYS36	1.92	HN-O	15
Kariya	293–337	GLU326—THR37	1.48	HN-O	10
Kasturi	293–337	SER323—ARG33	2.35	HN-O	17
Kavali kannu	112–156	PRO137—THR111	1.21	HN-O	14
Kulanji pille	253–297	ARG296—ALA129	1.12	HN-O	18
LD-43 (HLR-144)	313–357	LEU327 –SER9	1.13	HN-O	17
Mote bangarkaddi	181–225	ALA189—ILE20	1.41	HN-O	12
MTU 4870	296–340	GLU299—ILE10	1.41	HN-O	13
MTU-1061	293–337	VAL298—SER9	1.31	HN-O	12
ND 118	183–227	VAL201—SER9	1.42	HN-O	15
Orugallu	267–311	LYS275—CYS35	1.43	HN-O	16
Pant sankar dhan 1	293–337	GLN326—ASN135	0.98	HN-O	12
Pant sugandh dhan 17	266–309	CYS303—GLN2	1.03	HN-O	10
Parimala kalvi	313–357	TRP331 –TYR124	1.12	HN-O	17
PR 118	112–156	ALA148—LEU30	1.53	HN-O	18
Pusa basmati 1	313–357	ILE339—ALA5	1.53	HN-O	13
Pusa Sugandh 3	293–337	ILE312 –ILE7	1.60	HN-O	16
Pusa Sugandh 4	293–337	TYR298—THR28	2.16	HN-O	14
Sadabahar	313–357	GLY332—ILE7	1.76	HN-O	15
Samleshwari	112–156	GLU170 –GLN2	1.79	HN-O	18
Sanna mullare	181–225	ILE212 –ILE8	1.86	HN-O	14
Suphala	313–357	ILE345– GLN2	1.80	HN-O	11
Tadukan	130–174	CYS145 –LEU11	1.57	HN-O	16
Thule ate	181–225	GLU191—ALA51	1.58	HN-O	15
Tilak chandan	293–337	CYS301 –THR13	1.59	HN-O	14
Tiyun	290–309	THR305—THR4	1.31	HN-O	17
V L Dhan	112–156	SER144—ASN50	1.10	HN-O	17
Vanasurya	181–225	SER224—ALA32	1.05	HN-O	15
Varalu	313–357	ILE321 –GLN2	1.88	HN-O	17
Varun dhan	250–294	GLY255—ASN50	1.12	HN-O	10

### Binding free energy of Pi54: Avr-Pi54 proteins

The calculated binding energies for Pi54-AvrPi54 protein complexes for all protein pairs are given in the Table 7. The binding energy was found zero in cases where Pi54 protein did not interact with AvrPi54. There were 16 complexes with lesser binding energy than the Pi54^tetep^ protein, which involved proteins from the rice lines Casebatta, Tadukan, V L dhan, Varun dhan, Govind, Acharmita, Kavalikannu, HPR-2083, Budda, Jatto, MTU-4870, Dobeja-1, CN-1789, Indira sona, Kulanji pille and Mote bangarkaddi cultivars. These proteins thus have stronger interaction than the Pi54^tetep^ with the cognate partner AvrPi54 protein of the *M*. *oryzae*.

**Table 7:** The binding energy for interaction of AvrPi54 protein with the Pi54 proteins pairs.

Rice lines	Binding Energy	Rice lines	Binding Energy
Pi54 (Tetep)	-3835.00	Kariya	-2764.13
Acharmita	-4105.00	Kasturi	-2764.13
Basmati 386	-3643.58	Kavali kannu	-4015.00
Belgaum basmati	-2764.13	Kulanji pille	-3850.77
Bidarlocal-2	-3295.00	LD-43 (HLR-144)	-1802.77
Budda	-3980.19	Mote bangarkaddi	-3850.77
Casbatta	-4302.89	MTU 4870	-3980.19
Chiti zhini	-1762.09	MTU-1061	-3588.15
CN-1789	-3850.77	ND 118	-3745.00
CSR 10	-2764.13	Orugallu	-3682.71
CSR-60	-2764.13	Pant sankar dhan 1	-2009.77
Dobeja-1	-3925.00	Pant sankar dhan 17	-3817.33
Gonrra bhog	-2959.16	Parimala kalvi	-1318.96
Govind	-4202.68	PR 118	-3385.00
Gowrisanna	-2764.13	Pusa basmati 1	-1968.48
Himalya 799	-3778.33	Pusa Sugandh 3	-2009.77
HLR-108	-2552.63	Pusa Sugandh 4	-2764.13
HLR-142	-2419.67	Sadabahar	-2959.16
HPR 2083	-3986.22	Samleshwari	-3205.00
HPR-2178	-3115.00	Sanna mullare	-3565.00
IC356437	-2764.13	Suphala	-1968.48
Indira sona	-3850.77	Tadukan	-4302.89
Indrayani	-3790.43	Thule ate	-1968.48
INRC 779	-3655.00	Tilak chandan	-2764.13
IR 64	-3810.73	Tiyun	-3512.26
IRAT-144	-1968.48	V L Dhan	-4291.66
IRBB 55	-1289.09	Vanasurya	-3475.00
IRBB-13	-2009.77	Varalu	-2939.47
IRBB-4	-840.09	Varun dhan	-4240.00
Jatto	-3980.19	Varun dhan	-613.72
Kari kantiga	-2764.13		

### Molecular dynamic simulation

**Fig 5:**
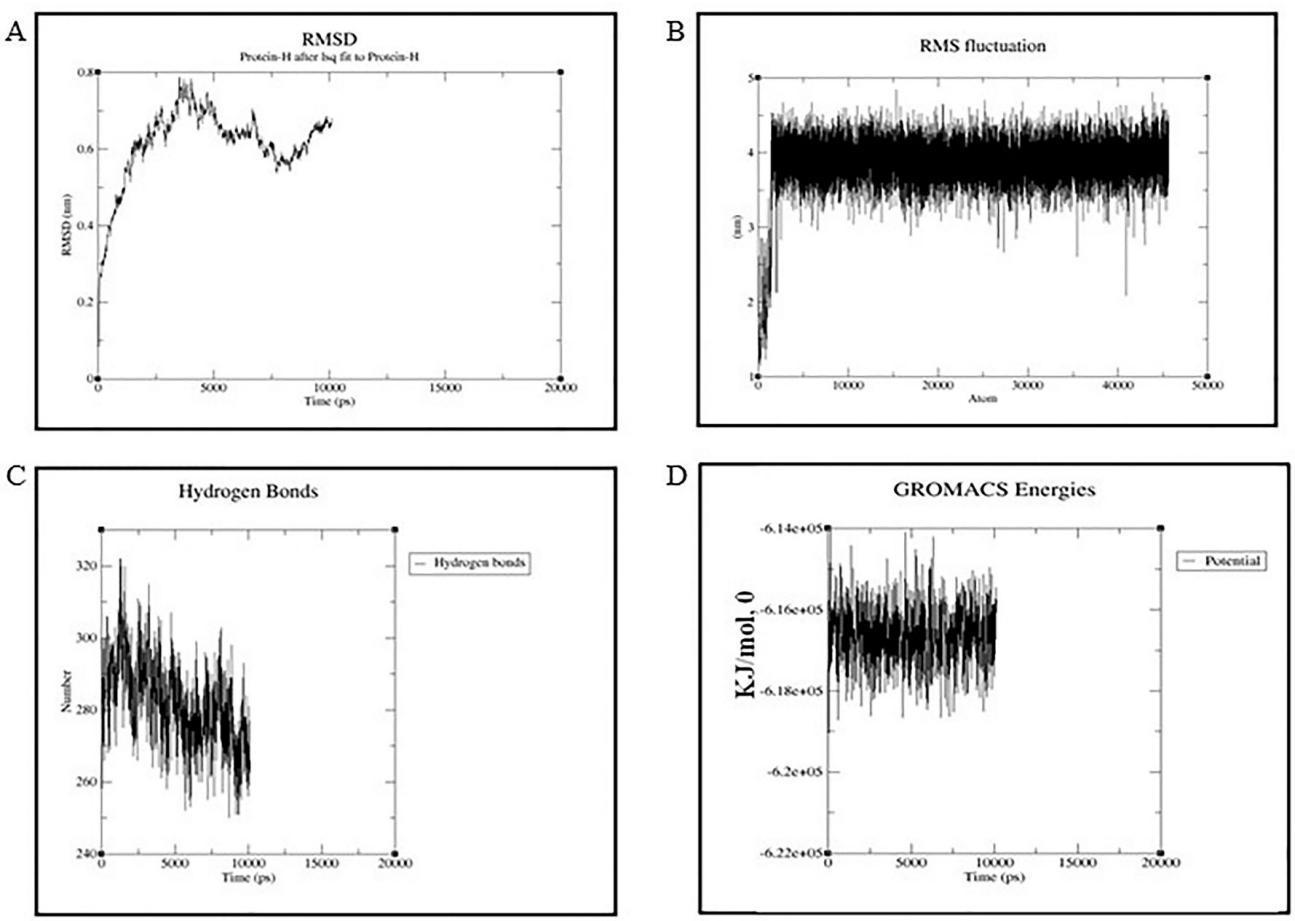
Molecular dynamics simulation of AvrPi54-Pi54^tetep^ protein complex. The complex acquired a stable conformation during the simulation trajectory, after deviating for about 1 Å in the first ns. Most of the peaks of RMSF plot possess a value between 0.1 and 0.4 nm. Thus favourable changes were achieved in the simulation process of docked AvrPi54 protein and the Pi54 proteins. **(A)** The Root Mean Square Deviation (RMSD), **(B)** Root Mean Square Fluctuation (RMSF), **(C)** Hydrogen bonds, and **(D)** Gyration energy plots.

Simulation is used for minimization of the energy and assessed the stability of the docked proteins complex. The complex acquired a stable conformation during the simulation trajectory, after deviating for about 1 Å in the first ns. The RMSD and RMSF for each residue of the complex were computed. RMSD is the global measure of fluctuations and is used to access the dynamic stability of the complex and RMSF is the time-average of RMSD for each residue. The number of Hydrogen bonds throughout simulations between the protein pair exhibited nominal change (Fig 5) showing that the complex remained stable during the rest of trajectory. Most of the peaks of RMSF plot possess a value between 0.1 and 0.4 nm. Thus favourable changes were achieved in the simulation process of docked AvrPi54 protein and the Pi54 proteins.

## Discussion

Natural variations harboured in the wild species, landraces and traditional varieties are the source of many agronomically important traits that furnish genes conferring resistance to diseases and pests and adaptation to environmental stresses. Disease resistance alleles from crop gene pool can be introgressed into high yielding varieties to further improve their capacity to overcome the challenges of pathogen attack [34]. Rice blast disease can be effectively managed by the utilisation of *R* gene which can be taken from the resistant varieties to develop high yielding varieties either by breeding or in raising transgenics [35]. The copy number variation, nucleotide diversity, substitutions at the *R* genes loci are sources of allelic variation [17,36]. *R* genes may harbour high or low levels of polymorphism [37]. The genome wide SNPs (Single Nucleotide Polymorphism) in these genes were also discovered in multiple rice genotypes [38]. When our docking results were compared to the phenotyping results from earlier studies [39], it was found that the interacting proteins belonged to the resistant rice line while the non-interacting proteins were from the susceptible rice lines (Table 8).

**Table 8:** Categorisation of the Pi54 proteins into resistant and susceptible lines.

S. No.	Rice line	Phenotype	S. No.	Rice line	Phenotype
1	Acharmati	R	35	Malviya dhan	S
2	Basmati 386	R	36	Mesebatta	S
3	Belgaum basmati	R	37	Mote bangarkaddi	R
4	Bidarlocal-2	R	38	MTU-1061	R
5	Budda	R	39	Orugallu	R
6	Chiti zhini	R	40	Pant sankar dhan 1	R
7	CN-1789	R	41	Pant sugandh dhan 17	R
8	CO-39	S	42	Parijat	S
9	CSR 10	R	43	Parimala kalvi	R
10	CSR-60	R	44	Pi54 (Tetep)	R
11	Gonrra bhog	R	45	PR 118	R
12	Gowrisanna	R	46	Pusa basmati 1	S
13	Himalya 799	R	47	Pusa Sugandh 3	R
14	HLR-108	R	48	Ram Jawain 100	S
15	HLR-142	R	49	Ranbir basmati	R
16	HPR 2083	R	50	Sadabahar	R
17	HPR-2178	R	51	Samleshwari	R
18	HR-12	S	52	Sanna mullare	R
19	Indira sona	R	53	Sathia -2	S
20	Indrayani	R	54	Satti	S
21	INRC 779	R	55	Shiva	S
22	IR 64	R	56	Superbasmati	S
23	IRAT-144	R	57	Suphala	R
24	IRBB 55	R	58	T23	S
25	IRBB-13	R	59	Tadukan	R
26	IRBB-4	R	60	Taipei-309	S
27	Jatto	R	61	Thule atte	R
28	Kari kantiga	R	62	Tilak chandan	R
29	Kariya	R	63	Tiyun	R
30	Kavali kannu	S	64	V L Dhan 21	R
31	Kulanji pille	R	65	Vanasurya	R
32	Lalnakanda	S	66	Varalu	R
33	HLR-144	R	67	Varun dhan	R
34	Mahamaya	R			

*Pi54* alleles contain polymorphism at the nucleotide level and are intermediately diversified due to the evolutionary pressure from pathogen side. The transition type SNPs occur more frequently than the transversions in these sequences. The presence of InDels of variable sizes and SNPs at various sites result into amino acids substitutions at the protein level [39,40]. A great variation in the lengths of these proteins is seen which is due to the presence of InDels in the coding region. The changes in amino acid residues and size of the proteins have led to the variations in their molecular weights. These proteins showed a very little difference in average composition of amino acids. The average leucine percentage of the Pi54 proteins was similar to that of the leucine percentage of Pi54^tetep^ protein. Resistant Pi54 proteins contained nearly 0.7% higher leucine content than the susceptible proteins. Many variations were obtained in their physiochemical properties. Most of the proteins were acidic in nature as indicated by the pI values. GRAVY index was found positive in all the proteins of susceptible alleles except the protein derived from rice line HR-12. It was negative for 14 resistant proteins most of which were found to bind more strongly with AvrPi54 than Pi54^tetep^ protein. The negative (−) GRAVY scores indicate that these proteins are hydrophilic. The low GRAVY index of resistance proteins infers that these hydrophilic proteins have more residues available for the formation of intermolecular hydrogen bonds while interacting with the AvrPi54 protein. These forces are significant in association and stability of protein complexes [41,42]. Therefore, polymorphism in the nucleotide sequence of the alleles has led to the variation in the amino acid sequence, leading to the differences in the physiochemical properties of these proteins.

The AvrPi54 protein was found to directly interact with the LRR domain of the resistant Pi54 proteins. The direct interaction between Pi54 and AvrPi54 proteins has earlier been confirmed *in vitro* by using the Yeast-two-hybrid analysis and *in planta* Tobacco leaf infiltration assay [20]. Non-synonymous substitutions were observed in LRR region of Pi54 gene. LRR domains are known to be located at the carboxy termini of plant resistance proteins. The domain acquires barrel-like structure lined with parallel β-sheets in the inner surface and α-helical structures in the remaining region. These structural units are arranged in the manner so that the protein acquires a non- globular shape similar to the horse shoe structure and is responsible for the protein- binding functions of the proteins [43]. In our analysis, the LRR domain was more conserved in the proteins from resistant cultivars than the susceptible ones. The interaction of AvrPi54 protein might have been interrupted with the susceptible alleles due to the amino acids substitution in the LRR region which seems to prevent the proper folding of the interacting LRR region for interaction. Mutation in LRR region, which maintains gene-for-gene specificity, may increase or decrease the recognition specificity [44]. Several studies have reported the effect of mutation in LRR domain on recognition capability and ligand binding specificity of R proteins. Change in a single amino acid in the β-strand region of the LRRs of polygalacturonase-inhibiting proteins confers a new recognition capability and increases ligand specificity in *Phaseolus vulgaris* [45]. A mutation that substitutes the amino acid glutamate to lysine within the LRR domain of a resistance gene RPS5 of Arabidopsis causes reduction in the resistance potential of several *R* genes that conferred resistance against multiple bacterial and downy mildew diseases [46]. Similarly, in the case of rice-*M*. *oryzae* pathosystem, a single amino acid change in the xxLxLxx motif of R proteins altered the surface through which they interact with their cognate Avr proteins [47].

The folding of the protein to its native structure is driven by various forces such as Hydrogen bonds that pack the helices and strands; Vander Waals interactions that tightly pack the atoms within a folded protein and the backbone angle preferences [48]. The secondary structure of the Pi54 proteins contained more number of turns and H-bonds in resistant cultivars. Numbers of α-helices were same in both resistant and susceptible proteins but the numbers of β-strands were found higher in case of resistance proteins. These factors could be accountable for greater stability of structures of resistance alleles in comparison with the susceptible alleles and also proper folding of the LRR region into protein interacting domain.

The occurrence of SNPs and InDels has created differences in the protein structures which gave rise to the differences in the interaction or interacting strength of Pi54 proteins with AvrPi54 protein. In this study, we found that some of the Pi54 proteins were structurally not similar to the Pi54^tetep^ protein. Though the Pi54 proteins had sequence similarity but these have very less residual similarity in three dimensional spatial arrangements. The similarity in the LRR region of Pi54 proteins of resistance alleles with the Pi54^tetep^ protein could be one of the reasons to allow successful interaction with the AvrPi54 protein. Binding energy for the Pi54 and Avr-Pi54 proteins also varied due to the structure variation among these proteins. The binding energy is a dependent variable on the global free minimum energy of the proteins. More number of H-bonds was found to minimize the binding energy of proteins. Out of 59 resistant proteins, only 15 resistant proteins from the land races: the Casebatta, Tadukan, VL Dhan, Varun dhan, Govind, Acharmita, HPR-2083, Budda, Jatto, MTU-4870, Dobeja-1, CN-1789, Indira sona, Kulanji pille and Motebangarkaddi were observed to show lower binding free energy with Avr-Pi54 proteins as compared to the Pi54^tetep^ protein, thus a stronger interaction potential.

## Conclusion

Our studies show that the nucleotide polymorphism in the Pi54 alleles is the cause of variations in physiochemical properties, LRR domain structure, protein structure, global free minimum energy, H-bond and global protein structures of both the resistant and susceptible alleles. These variations also affect the Pi54 and AvrPi54 interactions. The proteins from the resistant land races like Casebatta, Tadukan, VL Dhan, Varun dhan, Govind, Acharmita, HPR-2083, Budda, Jatto, MTU-4870, Dobeja-1, CN-1789, Indira sona, Kulanji pille and Mote bangarkaddi show stronger bonds with AvrPi54 protein than the Pi54^tetep^ protein. The resistant response would escalate if the interaction of the R and Avr partners is stronger [49]. Therefore, these alleles have more potential than the original resistance allele and can be effectively used for the rice blast resistance breeding program in future.

## Supporting information

S1 Table List of *Pi54* alleles used and their source rice lines. (PDF)

S1 Fig Composition of different amino acids in Pi54 proteins of the alleles cloned from resistant and susceptible rice lines compared with wild type Pi54^tetep^ protein. (PDF)

S2 Fig Three dimensional structures of various Pi54 proteins from rice lines. (PDF)

S3 Fig Docking images showing interaction of AvrPi54 protein and Pi54 proteins from rice lines. (PDF)

## References

[1] Wilson RA, Talbot NJ (2009) Under pressure: investigating the biology of plant infection by *Magnaporthe oryzae*. Nature Revi Microbiol 7: 185. 10.1038/nrmicro2032 19219052

[2] Fisher MC, Henk DA, Briggs CJ, Brownstein JS, Madoff LC, McCraw SL et al (2012) Emerging fungal threats to animal, plant and ecosystem health. Nature 484: 186 10.1038/nature10947 22498624 PMC3821985

[3] Sharma TR, Rai AK, Gupta SK, Vijayan J, Devanna BN, Ray S (2012) Rice blast management through host-plant resistance: retrospect and prospects. Agricult Res 1: 37–52.

[4] Fukuoka S, Yamamoto SI, Mizobuchi R, Yamanouchi U, Ono K, Kitazawa N, et al (2014) Multiple functional polymorphisms in a single disease resistance gene in rice enhance durable resistance to blast. Scientific Rep 4: 4550.

[5] Ma J, Lei C, Xu X, Hao K, Wang J, Cheng Z et al (2015) Pi64, encoding a novel CC-NBS-LRR protein, confers resistance to leaf and neck blast in rice. Mol Plant-Microbe Interact 28: 558–568. 10.1094/MPMI-11-14-0367-R 25650828

[6] Sirisathaworn T, Srirat T, Longya A, Jantasuriyarat C (2017) Evaluation of mating type distribution and genetic diversity of three Magnaporthe oryzae avirulence genes, PWL-2, AVR-Pii and Avr-Piz-t, in Thailand rice blast isolates. Agricult Nat Resour 51: 7–14.

[7] Flor HH (1971) Current status of the gene-for-gene concept. Annu Rev Phytopathol 9: 275–296.

[8] Dangl JL, Jones JD (2001) Plant pathogens and integrated defence responses to infection. Nature 411: 826 10.1038/35081161 11459065

[9] Zipfel C, Rathjen JP (2008) Plant immunity: AvrPto targets the frontline Curr Biol 18: 218–220. 10.1016/j.cub.2008.01.016 18334200

[10] Khush GS, Jena KK (2009) Current status and future prospects for research on blast resistance in rice (Oryza sativa L) In Advances in genetics, genomics and control of rice blast disease. 1–10 Springer, Dordrecht.

[11] Sharma TR, Madhav MS, Singh BK, Shanker P, Jana TK, Dalal V, et al (2005) High-resolution mapping, cloning and molecular characterization of the Pi-k h gene of rice, which confers resistance to *Magnaporthe griseae*. Mol Genet Genomics 274: 569–578. 10.1007/s00438-005-0035-2 16228246

[12] Sharma TR, Rai AK, Gupta SK, Singh NK (2010) Broad-spectrum blast resistance gene Pi-kh cloned from rice line Tetep designated as *Pi54*. J Plant Biochem Biotechnol 19: 87–89.

[13] Rai AK, Kumar SP, Gupta SK, Gautam N, Singh NK, Sharma TR (2011) Functional complementation of rice blast resistance gene Pi-k h (Pi54) conferring resistance to diverse strains of *Magnaporthe oryzae*. J Plant Biochem Biotechnol 20: 55–65.

[14] Gupta SK, Rai AK, Kanwar SS, Chand D, Singh NK, Sharma TR (2011) The single functional blast resistance gene Pi54 activates a complex defence mechanism in rice. J Exp Bot 63: 757–772. 10.1093/jxb/err297 22058403

[15] Gupta SK, Rai AK, Kanwar SS, Sharma TR (2012) Comparative analysis of zinc finger proteins involved in plant disease resistance. PLOS One 7: Se42578. 10.1371/journal.pone.0042578 PMC3419713 22916136

[16] Takken FLW, Tameling WIL (2009) To nibble at plant resistance proteins. Science 324: 744–746. 10.1126/science.1171666 19423813

[17] Thakur S, Singh PK, Rathour R, Variar M, Prashanthi SK, Singh AK et al (2013) Positive selection pressure on rice blast resistance allele Piz-t makes it divergent in Indian land races. J Plant Interact 8: 34–44.

[18] Das A, Soubam D, Singh PK, Thakur S, Singh NK, Sharma TR (2012) A novel blast resistance gene, *Pi54rh* cloned from wild species of rice, *Oryza rhizomatis* confers broad spectrum resistance to *Magnaporthe oryzae*. Funct Integr Genomics 12: 215–228. 10.1007/s10142-012-0284-1 22592658

[19] Devanna NB, Vijayan J, Sharma TR (2014) The blast resistance gene Pi54of cloned from *Oryza officinalis* interacts with Avr-Pi54 through its novel non-LRR domains. PLOS One 9: e104840 10.1371/journal.pone.0104840 25111047 PMC4128725

[20] Ray S, Singh PK, Gupta DK, Mahato AK, Sarkar C, Rathour R, et al, (2016) Analysis of Magnaporthe oryzae genome reveals a fungal effector, which is able to induce resistance response in transgenic rice line containing resistance gene, Pi54. Front Plant Sci 7, 1140 10.3389/fpls.2016.01140 27551285 PMC4976503

[21] Yavuz C, Öztürk ZN (2017) Working with Proteins in silico: A Review of Online Available Tools for Basic Identification of Proteins Turk J Agri -Food Sci Technol 5: 65–70.

[22] Meng XY, Zhang HX, Mezei M, Cui M (2011) Molecular docking: a powerful approach for structure-based drug discovery. Current Comput-aided Drug Des 7: 146–157. 10.2174/157340911795677602 PMC3151162 21534921

[23] Ehrenfeld N, Gonzalez A, Canon P, Medina C, Perez-Acle T, Arce-Johnson P (2008) Structure–function relationship between the tobamovirus TMV-Cg coat protein and the HR-like response. J Gen Virol 89: 809–817. 10.1099/vir.0.83355-0 18272773

[24] Rozas J and Rozas R (1995) DnaSP, DNA sequence polymorphism: an interactive program for estimating population genetics parameters from DNA sequence data. Bioinformatics 11: 621–625 10.1093/bioinformatics/11.6.621 8808578

[25] Waterhouse AM, Procter JB, Martin DM, Clamp M and Barton GJ, 2009 Jalview Version 2—a multiple sequence alignment editor and analysis workbench Bioinformatics 25: 189–1191. 10.1093/bioinformatics/btp033 PMC2672624 19151095

[26] Berman HM, Westbrook J, Feng Z, Gilliland G, Bhat TN, Weissig H et al (2000) The protein data bank. Nucleic Acids Res 28: 235–242. 10.1093/nar/28.1.235 10592235 PMC102472

[27] Altschul SF, Gish W, Miller W, Myers EW, Lipman DJ (1990) Basic local alignment search tool. J Mol Biol 215: 403–410. 10.1016/S0022-2836(05)80360-2 2231712

[28] Sayle RA, Milner-White EJ (1995) RASMOL: biomolecular graphics for all Trends. Biochem Sci 20: 374–376. 10.1016/s0968-0004(00)89080-5 7482707

[29] Ramachandran GT, Sasisekharan V (1968) Conformation of polypeptides and proteins. Adv Protein Chem 23: 283–437. 4882249 10.1016/s0065-3233(08)60402-7

[30] Brooks BR (1983) A program for macromolecular energy, minimization, and dynamics calculations. J Comput Chem 4: 187–217.

[31] Abraham MJ, Murtola T, Schulz R, Páll S, Smith JC, Hess B, et al (2015) GROMACS: High performance molecular simulations through multi-level parallelism from laptops to supercomputers. SoftwareX 1: 19–25.

[32] Schmid N, Eichenberger AP, Choutko A, Riniker S, Winger M, Mark AE et al (2011) Definition and testing of the GROMOS force-field versions 54A7 and 54B7. Eur Biophys J 40: 843 10.1007/s00249-011-0700-9 21533652

[33] Ikai A (1980) Thermostability and aliphatic index of globular proteins. J Biochem 88: 1895–1898. 7462208

[34] Singh PK, Thakur S, Rathour R, Variar M, Prashanthi SK, Singh AK, et al (2014) Transposon-based high sequence diversity in Avr-Pita alleles increases the potential for pathogenicity of *Magnaporthe oryzae* populations. Funct Integr Genomics 14: 419–429. 10.1007/s10142-014-0369-0 24633351

[35] Singh PK, Nag A, Arya P, Kapoor R, Singh A, Jaswal R et al (2018) Prospects of Understanding the Molecular Biology of Disease Resistance in Rice. Int J Mol Sci 19: 1141. 10.3390/ijms19041141 PMC5979409 29642631

[36] Thakur S, Gupta YK, Singh PK, Rathour R, Varia M, Prashanthi SK et al (2013) Molecular diversity in rice blast resistance gene Pi-ta makes it highly effective against dynamic population of *Magnaporthe oryzae*. Funct Integr Genomics 13: 309–322. 10.1007/s10142-013-0325-4 23818197

[37] Yang S, Gu T, Pan C, Feng Z, Ding J, Hang Y, et al (2008) Genetic variation of NBS-LRR class resistance genes in rice lines. Theor Appl Genet 1:165–177. 10.1007/s00122-007-0656-4 17932646

[38] Yamamoto T, Nagasaki H, Yonemaru JI, Ebana K, Nakajima M, Shibaya T et al (2010) Fine definition of the pedigree haplotypes of closely related rice cultivars by means of genome-wide discovery of single-nucleotide polymorphisms. BMC genomics 11: 267 10.1186/1471-2164-11-267 20423466 PMC2874813

[39] Thakur S., Singh P.K., Das A., Rathour R., Variar M., Prashanthi S.K., Singh A.K., Singh U.D., Chand D., Singh N.K. and Sharma T.R., 2015 Extensive sequence variation in rice blast resistance gene Pi54 makes it broad spectrum in nature. Frontiers in plant science, 6: 345 10.3389/fpls.2015.00345 26052332 PMC4440361

[40] Singh PK, Ray S, Thakur S, Rathour R, Sharma V Sharma TR (2018) Co-evolutionary interactions between host resistance and pathogen avirulence genes in rice-*Magnaporthe oryzae* pathosystem. Fungal Genet Biol 115, 9–19 10.1016/j.fgb.2018.04.005 29630984

[41] Jiang L, Lai L (2002) CH··· O hydrogen bonds at protein-protein interfaces. J Biol Chem 277: 37732–37740. 10.1074/jbc.M204514200 12119293

[42] Eildal JN, Hultqvist G, Balle T, Stuhr-Hansen N, Padrah S, Gianni S et al (2013) Probing the role of backbone hydrogen bonds in protein–peptide interactions by amide-to-ester mutations. J Am Chem Soc 135: 12998–13007. 10.1021/ja402875h 23705582

[43] Kobe B, Deisenhofer J (1994) The leucine-rich repeat: a versatile binding motif. Trends Biochem Sci 19: 415–421. 10.1016/0968-0004(94)90090-6 7817399

[44] Wulff BB, Thomas CM, Smoker M, Grant M, Jones JD (2001) Domain swapping and gene shuffling identify sequences required for induction of an Avr-dependent hypersensitive response by the tomato Cf-4 and Cf-9 proteins. Plant Cell 13: 255–272. 10.1105/tpc.13.2.255 11226184 PMC102241

[45] Leckie F, Mattei B, Capodicasa C, Hemmings A, Nuss L, Aracri B, et al (1999) The specificity of polygalacturonase‐inhibiting protein (PGIP): a single amino acid substitution in the solvent‐exposed β‐strand/β‐turn region of the leucine‐rich repeats (LRRs) confers a new recognition capability. EMBO J 18: 2352–2363. 10.1093/emboj/18.9.2352 10228150 PMC1171318

[46] Warren RF, Henk A, Mowery P, Holub E, Innes RW (1998) A mutation within the leucine-rich repeat domain of the Arabidopsis disease resistance gene RPS5 partially suppresses multiple bacterial and downy mildew resistance genes. Plant Cell 10: 1439–1452. 10.1105/tpc.10.9.1439 9724691 PMC144076

[47] Zhou B, Qu S, Liu G, Dolan M, Sakai H, Lu G, et al 2006 The eight amino-acid differences within three leucine-rich repeats between *Pi2* and *Piz-t* resistance proteins determine the resistance specificity to *Magnaporthe grisea*. Mol Plant-Microbe Interact 19: 1216–28. 10.1094/MPMI-19-1216 17073304

[48] Dill KA, MacCallum JL (2012) The protein-folding problem, 50 years on Science. 338: 1042–1046. 10.1126/science.1219021 23180855

[49] Kanzaki H, Yoshida K, Saitoh H, Fujisaki K, Hirabuchi A, Alaux L, et al (2012) Arms race co‐evolution of *Magnaporthe oryzae* AVR‐Pik and rice Pik genes driven by their physical interactions. Plant J 72: 894–907. 10.1111/j.1365-313X.2012.05110.x 22805093

