# Supplementary material for: Variation in the LRR region of Pi54 protein alters its interaction with the AvrPi54 protein revealed by in silico analysis
Source: PLoS One. 2019 Nov 5;14(11):e0224088. doi: 10.1371/journal.pone.0224088 (PMC6830779; doi:10.1371/journal.pone.0224088)
Supplement: S1 Table — (PDF) [file pone.0224088.s001.pdf]

S1 Table. List of *Pi54* alleles used their source of rice lines

S. No.	Sample Name	Accession No.	Seq. Length
1.	Tetep	AY914077	1459
2.	Acharmati	HE586221	1935
3.	Basmati 386	HE586211	2240
4.	Belgaum basmati	HE586171	1690
5.	Bidarlocal-2	HE586165	1851
6.	Budda	HE586186	1730
7.	Casbatta	HE586160	1852
8.	Chiti zhini	HE586169	1695
9.	CN-1789	HE586205	1974
10.	CSR 10	HE586204	1793
11.	CSR-60	HE586208	1804
12.	Dobeja-1	HE586199	1754
13.	Gonrra bhog	HE586195	1810
14.	Govind	HE586242	2141
15.	Gowrisanna	-	1851
16.	Himalya 799	HE586209	1822
17.	HLR-108	HE586177	1657
18.	HLR-142	HE586176	1642
19.	HPR 2083	HE586240	1650
20.	HPR-2178	HE586167	1663
21.	HR-12	HE586159	1647
22.	IC356437	HE586178	1689

23.	Indira sona	HE586213	1797
24.	Indrayani	HE586180	1802
25.	INRC 779	HE586181	2022
26.	IR 64	HE586256	2018
27.	IRAT-144	HE586255	1628
28.	IRBB 55	HE586203	2236
29.	IRBB-13	HE586246	1781
30.	IRBB-4	HE586234	2173
31.	Jatto	HE589455 -	1471
32.	Kari kantiga	HE586174	1687
33.	Kariya	HE586170	1672
34.	Kasturi	HE586230	1805
35.	Kulanji pille	HE586190	1817
36.	Lalnakanda	HE586217	1815
37.	LD-43 (HLR-144)	HE586183	1763
38.	Mahamaya	HE586253	1792
39.	Malviya dhan	HE586251	1823
40.	Mesebatta	HE586173	1878
41.	Mote bangarkaddi	HE586184	1937
42.	MTU-1061	HE586196	2151
43.	MTU-4870	HE586218	2031
44.	ND-118	HE586201	1900
45.	Orugallu	HE586163	1466
46.	Pant sankar dhan 1	HE586243	1782
47.	Pant sugandh dhan 17	HE586215	1789

48.	Parijat	HE586225	1820
49.	Parimala kalvi	HE586172	1696
50.	PR 118	HE586252	1829
51.	Pusa basmati 1	HE586254	1787
52.	Pusa Sugandh 3	HE586249	1779
53.	Pusa sugandh 4	HE586248	1806
54.	Ram Jawain 100	HE586164	1466
55.	Ranbir basmati	HE586212	1816
56.	Sadabahar	HE586236	1771
57.	Samleshwari	HE586200	1956
58.	Sanna mullare	HE586188	1881
59.	Sathia -2	HE586182	1828
60.	Satti	HE586206	1781
61.	Shiva	HE586210	2601
62.	Superbasmati	HE586224	1804
63.	Suphala	HE586219	1813
64.	T23	HE586250	1804
65.	Tadukan	HE586156	1646
66.	Taipei-309	HE586157	1687
67.	Thule ate	HE586185	1756
68.	Tilak chandan	HE586207	1793
69.	Tiyun	HE586168	1674
70.	Vanasurya	HE586189	1956
71.	Varalu	HE586239	1809
72.	Varun dhan	HE586199	1754
