# Supplementary material for: Variation in the LRR region of Pi54 protein alters its interaction with the AvrPi54 protein revealed by in silico analysis
Source: PLoS One. 2019 Nov 5;14(11):e0224088. doi: 10.1371/journal.pone.0224088 (PMC6830779; doi:10.1371/journal.pone.0224088)

Fig S1. Composition of different amino acids in Pi54 proteins of the alleles cloned from resistant and susceptible rice lines compared with wild type Pi54tetep protein

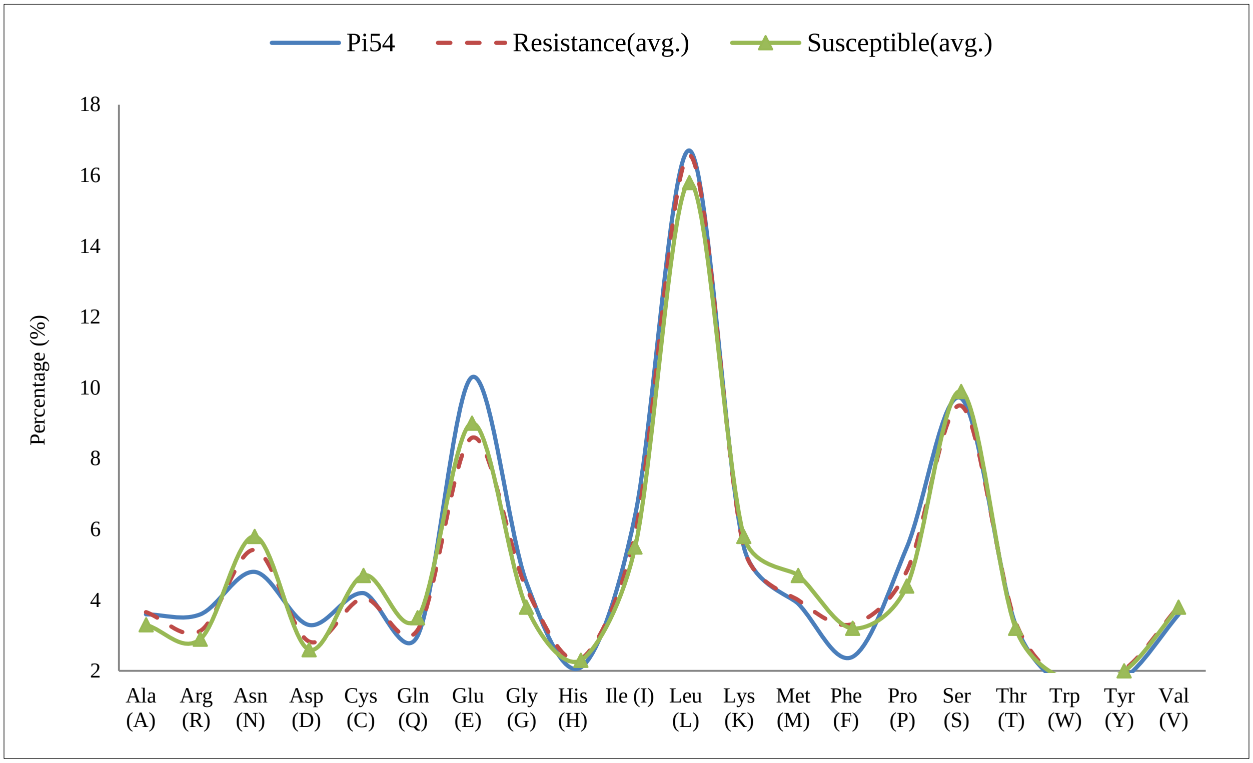

Supplement: S1 Fig — (PDF) [file pone.0224088.s002.pdf]
