# Supplementary material for: Variation in the LRR region of Pi54 protein alters its interaction with the AvrPi54 protein revealed by in silico analysis
Source: PLoS One. 2019 Nov 5;14(11):e0224088. doi: 10.1371/journal.pone.0224088 (PMC6830779; doi:10.1371/journal.pone.0224088)
Supplement: S2 Fig — (PDF) [file pone.0224088.s003.pdf]

S2 Fig. Three dimensional structures of Pi54 proteins from rice lines. A-Acharmati, BBasmati 386, C- Belgaum basmati, D-Bidarlocal-2, E-Budda, F-Chiti zhini, G- CN-1789, HCSR 10, I-CSR-60, J- Dobeja-1, K-Gonrra bhog, L- Govind, M- Gowrisanna, N- Himalya 799, O- HLR-108, P- HLR-142, Q- HPR-2178, R- HR-12, S- IC356437, T- Indira sona, UIndrayani, V- INRC 779, W- IR 64, X- IRAT-144, Y- IRBB 55, Z- IRBB-13, a- IRBB-4, bJatto , c-Kari kantiga, d- Kariya, e-Kasturi, f- Kulanji pille, g-Lalnakanda, h- LD-43 (HLR-144), i- Mahamaya, j- Malviya dhan, k- Mesebatta, l- Mote bangarkaddi, m- MTU-1061, nMTU-4870, o- ND-118, p- Orugallu, q- Pant sankar dhan 1, r- Pant sugandh dhan 17, sParijat, t- Parimala kalvi, u- PR 118, v- Pusa basmati 1, w- Pusa Sugandh 3, x- Pusa sugandh 4, y- Ram Jawain 100, z- Ranbir basmati, A'- Sadabahar, B'- Samleshwari, C'- Sanna mullare, D'- Sathia -2, E'- Satti, F'- Shiva, G'- Superbasmati, H'- Suphala, I'- T23, J'- Tadukan, K'- Taipei-309, L'- Thule ate, M'- Tilak chandan, N'- Tiyun, O'- Vanasurya, P'- Varalu, Q'- Varun dhan

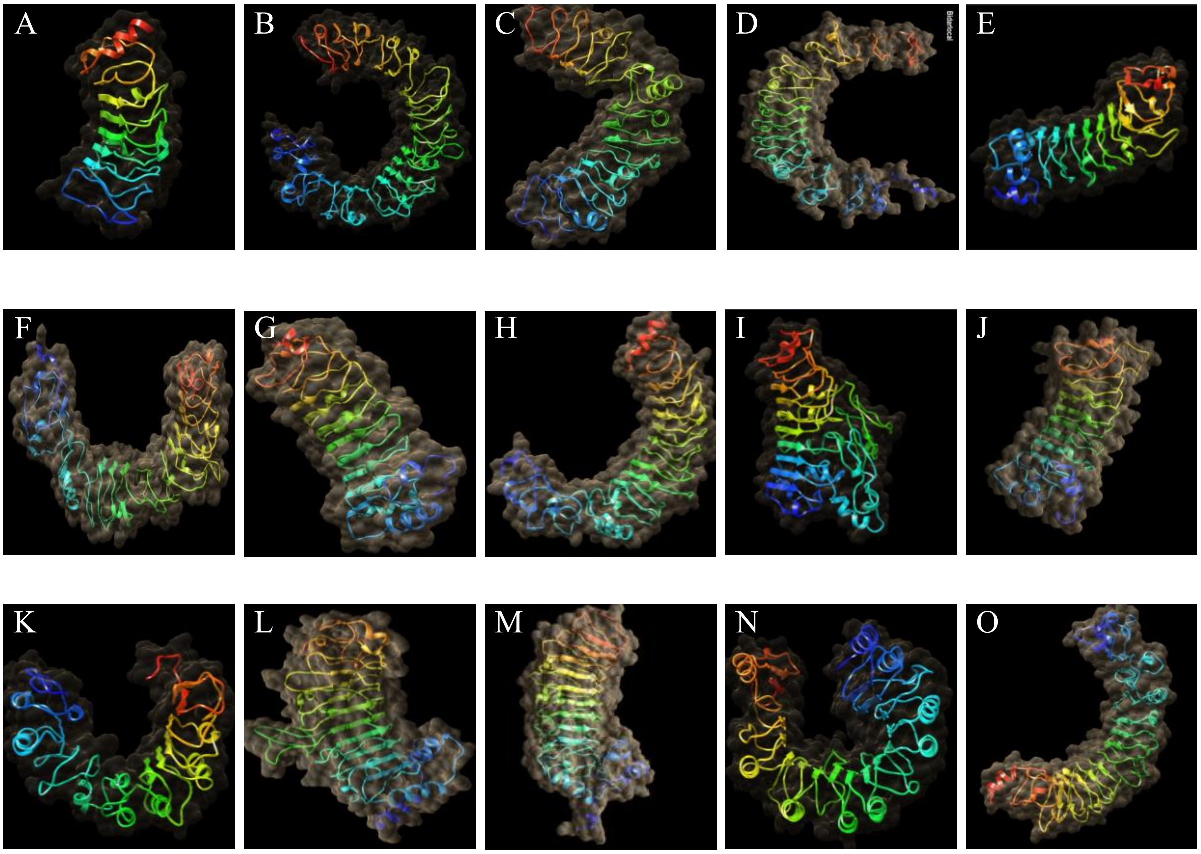

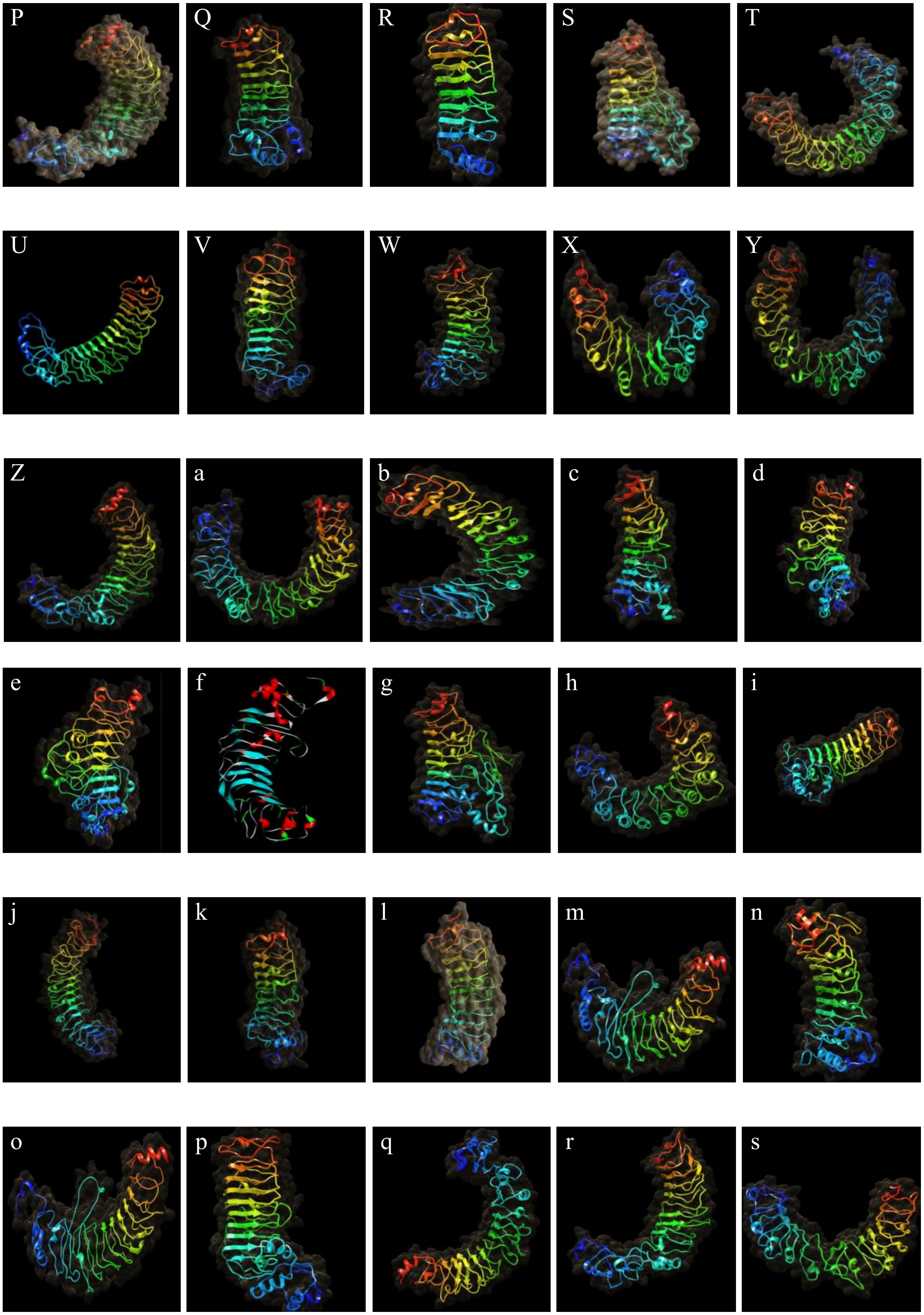
P
Q
R
S
T
U
V
W
X
Y
Z
a
b
c
d
e
f
g
h
i
j
k
l
m
n
o
p
q
r
s

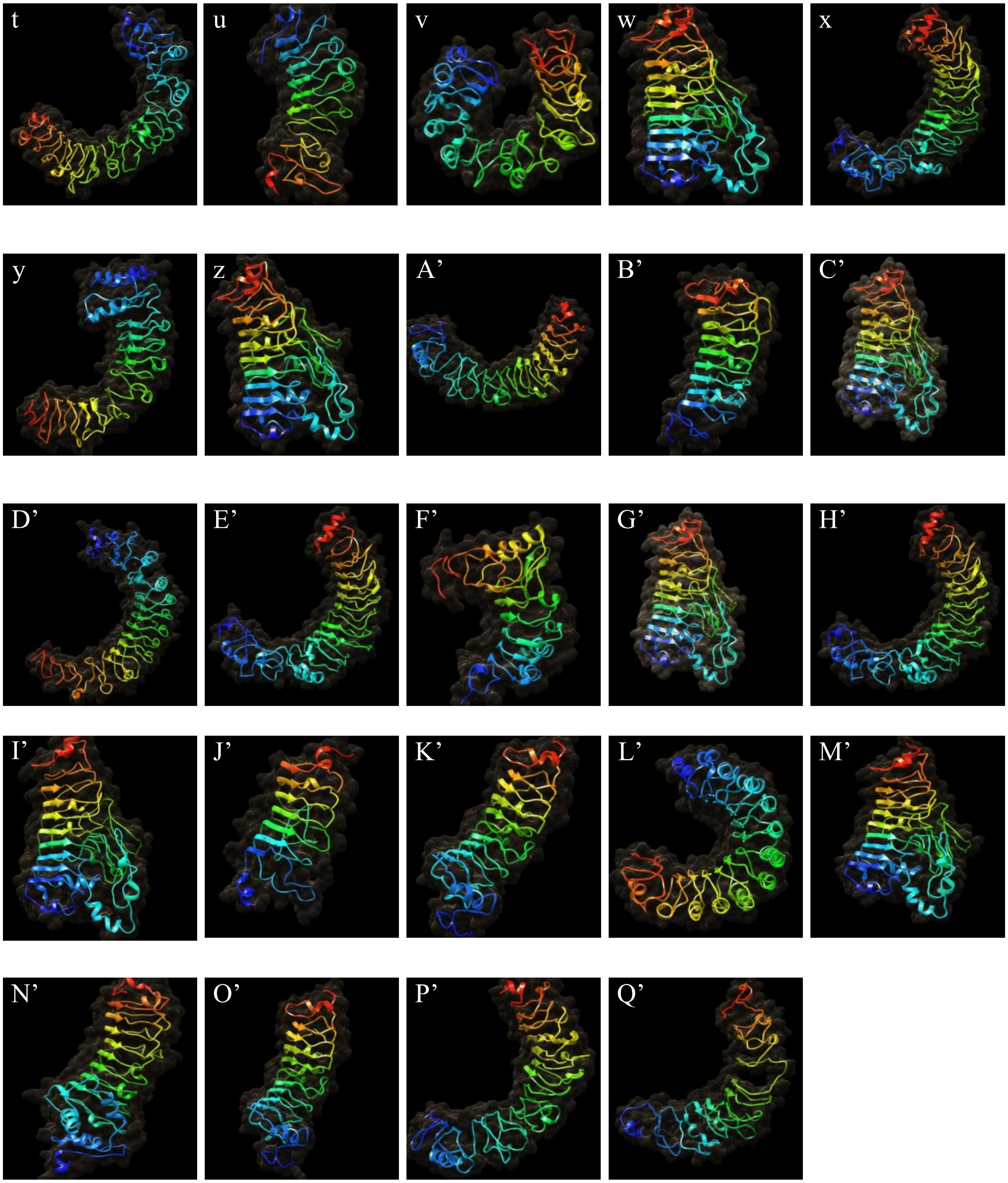
t
u
v
w
x
y
z
A’
B’
C’
D’
E’
F’
G’
H’
I’
J’
K’
L’
M’
N’
O’
P’
Q’
