# Supplementary material for: Variation in the LRR region of Pi54 protein alters its interaction with the AvrPi54 protein revealed by in silico analysis
Source: PLoS One. 2019 Nov 5;14(11):e0224088. doi: 10.1371/journal.pone.0224088 (PMC6830779; doi:10.1371/journal.pone.0224088)
Supplement: S3 Fig — (PDF) [file pone.0224088.s004.pdf]

S3 Fig. **Docking images showing interaction of Avr-Pi54 protein and Pi54 proteins from rice lines.** A-Acharmati, B- Basmati 386, C- Belgaum basmati, D-Bidarlocal-2, E-Budda, FChiti zhini, G-CN-1789, H-CSR 10, I-CSR-60, J- Dobeja-1, K-Gonrra bhog, L- Govind, MGowrisanna, N- Himalya 799, O- HLR-108, P- HLR-142, Q- HPR-2178, R- IC356437 , SIndira sona, T- Indrayani, U- INRC 779, V- IR 64, W- IRAT-144, X- IRBB 55, Y- IRBB-13, Z- IRBB-4, a- Jatto, b- Kari kantiga, c- Kariya, d- Kasturi, e- Kulanji pille, f- LD-43 (HLR-144), g- Mote bangarkaddi, h- MTU-1061, i- MTU-4870, j- ND-118, k- Orugallu, l- Pant sankar dhan 1, m- Pant sugandh dhan 17, n- Parimala kalvi, o- PR 118 , p- Pusa basmati 1, qPusa Sugandh 3, r- Pusa sugandh 4, s- Sadabahar, t- Samleshwari, u- Sanna mullare, vSuphala,w- T23, x- Tadukan, y- Thule ate, z- Tilak chandan, A'- Tiyun, B'- Vanasurya, C'- Varalu, D'- Varun dhan

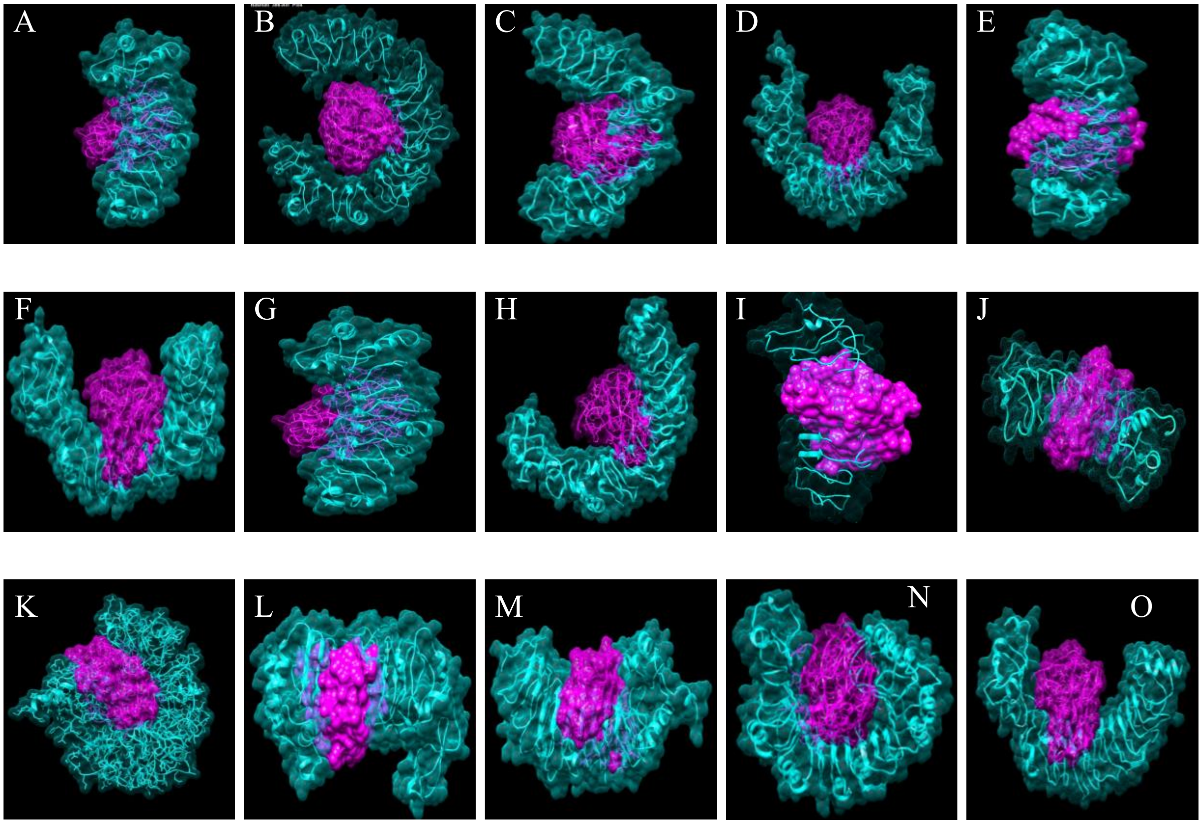

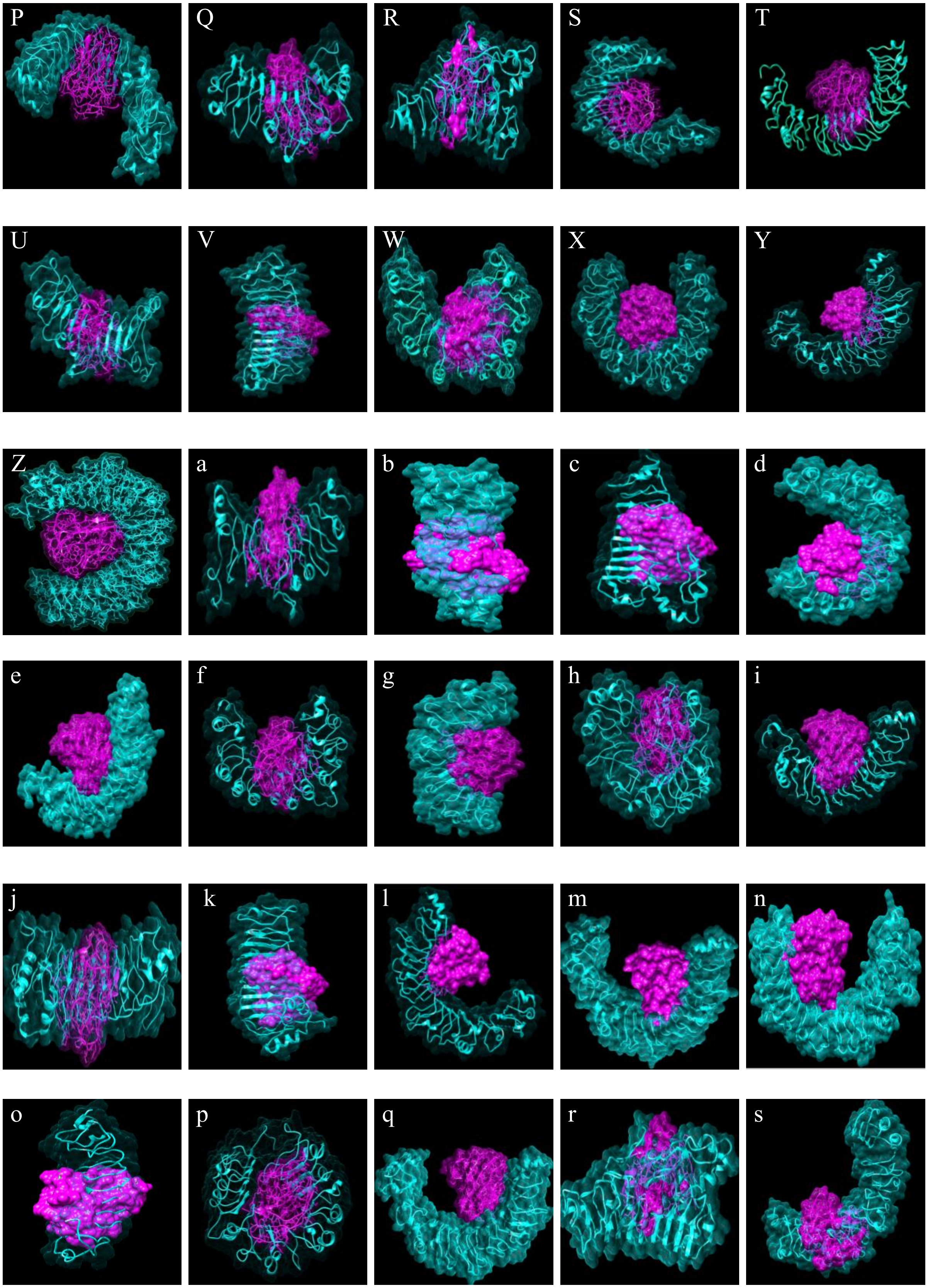
P
Q
R
S
T
U
V
W
X
Y
Z
a
b
c
d
e
f
g
h
i
j
k
l
m
n
o
p
q
r
s

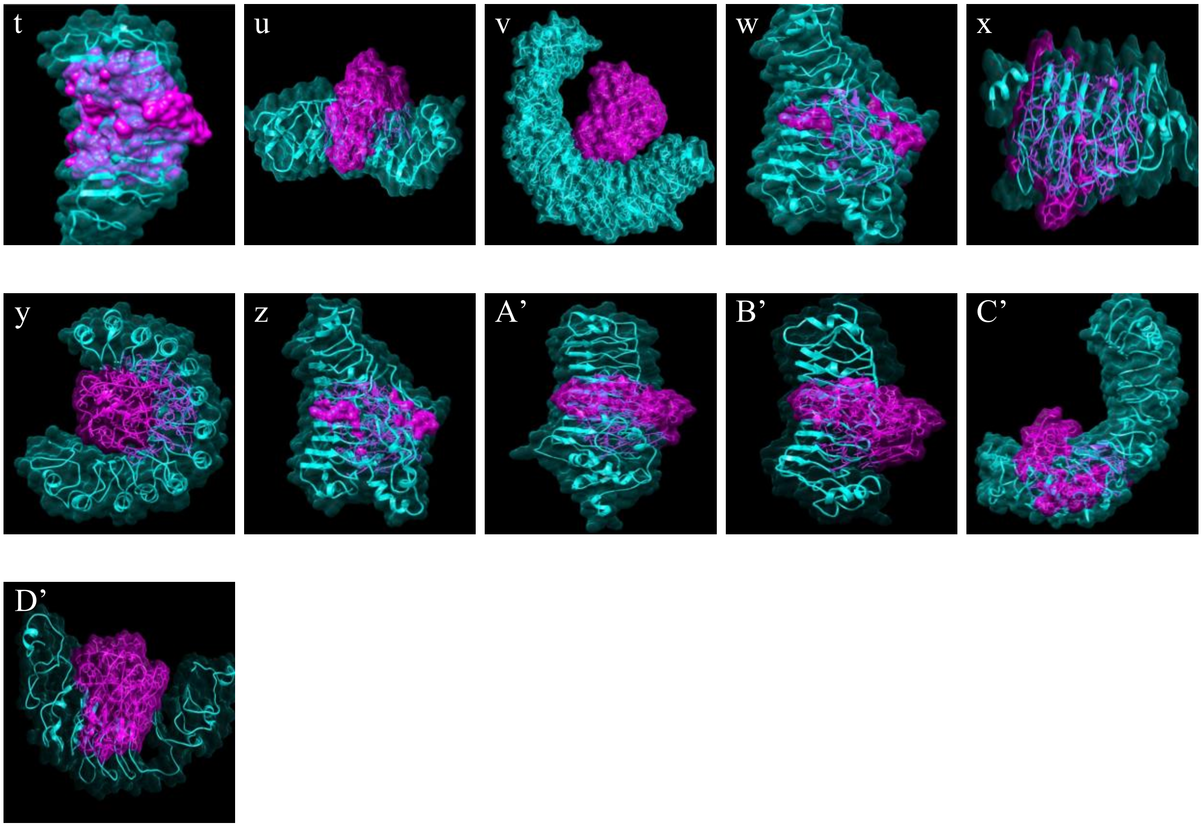
t
u
v
w
x
y
z
A’
B’
C’
D’
