# Supplementary material for: Variation in the LRR region of Pi54 protein alters its interaction with the AvrPi54 protein revealed by in silico analysis
Source: PLoS One. 2019 Nov 5;14(11):e0224088. doi: 10.1371/journal.pone.0224088 (PMC6830779; doi:10.1371/journal.pone.0224088)
Supplement: Attachment — Submitted filename: Reply to reviewers comments.docx [file pone.0224088.s005.docx]

**Reviewer 1** **comments**

The papers presents the results of the nucleotide polymorphisms in the Pi54 alleles which affect the Pi54 and AvrPi54 interaction. The title of the papers should be more specific since many of studies have been done on the R-Avr protein interaction including in the author papers published in 2014, which revealed that the Pi54of interacted with AVR-Pi54 through its non-LRR region, but Pi54 and Pi54rh interacted through CC-non-LRR-LRR regions and non-LRR-LRR regions, and the susceptible Pi54tp protein did not show interaction with AVR-Pi54, respectively. So this papers judged from the title only provides a study is similar to the former’.

The major problem of the papers is that the conclusions in the papers should be verified by experiment, the molecular simulation is not real happened after all. Furthermore, there are many problems with sentence structure and clause construction.

So try to set the problem discussed above in more detail!)

**Our reply**

Title of the manuscript is changed as per the suggestion of the reviewer that is “Variation in the LRR region of Pi54 protein alters its interaction with the AvrPi54 protein revealed by *in silico* analysis”.

The experimental proof for the interaction between Avr-Pi54 and Pi54 proteins was already done by Ray et al, 2016 ([Front Plant Sci](https://www.ncbi.nlm.nih.gov/pmc/articles/PMC4976503/). 2016; 7: 1140). Here, the main objectives of this manuscript was to analyse sequence variation at the LRR regions of those alleles of Pi54 gene isolated from both phenotypically resistant and susceptible rice lines and analysed sequence variation at the LRR region and look at their interaction with AvrPi54 gene. The hypothesis was that the proteins of Pi54 alleles isolated from resistant rice lines should show in silico interaction with the AvrPi54 proteins and the susceptible one should not show any interaction which is akin to the Flor`s gene-for-gene hypothesis (Flor 1972). The interaction analysis revealed that Pi54 protein from resistance lines, showed significant *in silico* interactions with AvrPi54 protein, while Pi54 from susceptible lines did not interact with its counterpart. Hence, it proves our hypothesis.

We made corrections in the conclusion section of the manuscript in lines 527 and 529. We also corrected many sentences throughout the manuscript, e.g. in lines 56, 71, 77, 83, etc. These changes can be seen in the track change version of the manuscript.

In addition in the revised version of the MS, molecular dynamics simulation analysis was carried out to see whether the interactions between Avr-Pi54 and Pi54 proteins are stable at the thermodynamics level or not, and found that the interaction was stable at the different thermodynamic parameters.

**Reviewer 2 comments**:

The authors analysed the Pi54 alleles from 72 rice lines by using bioinformatics methods to understand the interaction of Pi54 (R) proteins with AvrPi54 (Avr) protein. And they found that the physiochemical properties of these proteins varied due to the nucleotide level polymorphism. Generally, the authors present a well conducted study. The manuscript is concise and the results are mainly convincing.

**Some minor points:**

**1.** Figure 1, it can be hard to distinguish the 13 different amino acids. It may be useful to draw some lines.

**Our reply**

The figure 1 has been removed from the main text and instead of this figure a comparative composition of amino acids of the resistant and susceptible proteins with Pi54^tetep^ protein is given, but annexed to supplementary data as figure S1 titled as “Composition of different amino acids in Pi54 proteins of the alleles cloned from resistant and susceptible rice line (A) compared with wild type Pi54^tetep^ protein”.

**2.** Figure 2, add the letters to indicate which one is 2A or 2B.

**Our reply**

As suggested the changes have been made.

**3.** It could be better for this manuscript if the authors could do some Y2H to check the interactions between avrpi54 and Pi54 alleles from resistant and susceptible lines. These experimental results could provide stronger evidence to explain the different phenotype.

**Our reply**

The Y2H assay has been already performed between the AvrPi54 and Pi54^tetep^ protein to confirm their interaction by Ray et al, 2016, “Analysis of *Magnaporthe oryzae* genome reveals a fungal effector, which is able to induce resistance response in transgenic rice line containing resistance gene, Pi54” published in Frontiers in Plant Science journal and this publication has been cited in this manuscript. In this article, authors were identified the AvrPi54 gene and validated the gene using the *in vitro* as well as the *in planta* experiments. The significant interaction between the candidates of AvrPi54 and the Pi54 protein was initially begun with the bioinformatics approaches and led the analysis to pinpoint the *AvrPi54* gene. Thus we hypothesized that to check whether the phenotypic results of rice blast disease screening experiment, on *Pi54* resistant rice line and susceptible rice line would be matched with the results of *in silico* interaction between AvrPi54 and variants of Pi54 proteins. For that we conducted this study and disrupted interactions were found when variants of Pi54 derived from susceptible rice lines interacted with the AvrPi54 protein, while a strong interaction with AvrPi54 was found in case of Pi54 variants obtained from resistant rice lines. Thus, any of the Pi54 allele could be initially determined for its function through the *in silico* interaction analysis without doing phenotyping experiment. That is the main objective of this study. The interaction between Pi54-AvrPi54 proteins has been taken as a basis to extend our knowledge to discover the strength of interaction between various allelic Pi54 proteins with the AvrPi54 protein.
